# Proximity effects in the electron ionisation mass spectra of substituted cinnamamides

**DOI:** 10.1177/14690667231153777

**Published:** 2023-02-16

**Authors:** Adam R Michalik, Nathan W Fenwick, Richard Telford, Archie W Johnson, William HC Martin, Richard D Bowen

**Affiliations:** School of Chemistry and Biosciences, 123019 Faculty of Life Sciences, 1905 University of Bradford, Bradford, UK

**Keywords:** Electron ionisation, cinnamamides, proximity effects, cyclisation, isomerisation, substituent effects, simple cleavage

## Abstract

The electron ionisation mass spectra of an extensive set of 53 ionised monosubstituted and disubstituted cinnamamides [XC_6_H_4_CH=CHCONH_2_, X = H, F, Cl, Br, I, CH_3_, CH_3_O, CF_3_, NO_2_, CH_3_CH_2_, (CH_3_)_2_CH and (CH_3_)_3_C; and XYC_6_H_3_CH=CHCONH_2_, X = Y = Cl; and X, Y = F, Cl or Br] are reported and discussed. Particular attention is paid to the significance of loss of the substituent, X, from the 2-position, via a rearrangement that is sometimes known as a proximity effect, which has been reported for a range of radical-cations, but is shown in this work to be especially important for ionised cinnamamides. When X is in the 2-position of the aromatic ring, [M – X]^+^ is formed to a far greater extent than [M – H]^+^; in contrast, when X is in the 3-position or 4-position, [M – H]^+^ is generally much more important than [M – X]^+^. Parallel trends are found in the spectra of XYC_6_H_3_CH=CHCONH_2_: the signal for [M – X]^+^ dominates that for [M – Y]^+^ when X is in the 2-position and Y in the 4-position or 5-position, irrespective of the nature of X and Y. Further insight is obtained by studying the competition between expulsion of X^·^ and alternative fragmentations that may be described as simple cleavages. Loss of ^·^NH_2_ results in the formation of a substituted cinnamoyl cation, [XC_6_H_4_CH=CHCO]^+^ or [XYC_6_H_3_CH=CHCO]^+^; this process competes far less effectively with the proximity effect when X is in the 2-position than when it is in the 3-position or 4-position. Additional information has been obtained by investigating the competition between formation of [M – H]^+^ by the proximity effect and loss of CH_3_^·^ by cleavage of a 4-alkyl group to give a benzylic cation, [R^1^R^2^CC_6_H_4_CH=CHCONH_2_]^+^ (R^1^, R^2^ = H, CH_3_).

## Introduction

Despite incontrovertible evidence to the contrary, the myth that mass spectrometry can never furnish information on the substitution pattern of aromatic compounds is still widely believed by organic chemists. ‘*Ortho* effects’, in which two adjacent substituents in an ionised aromatic compound interact, are one class of rearrangement that may permit *ortho* disubstituted substrates to be distinguished from their *meta* and *para* isomers. Well-documented examples include loss of RZH (Z = O, NH; R = H, C_n_H_2n+1_) from ionised *ortho* substituted benzoic acid derivatives, equation (1),^1,2^ and ejection of HO^·^ from ionised nitroaromatic compounds containing an *ortho* methyl group, equation (2).^2^ In contrast, the isomeric radical-cations with no *ortho* substituent lose RZ^·^ and NO_2_^·^, respectively, as would be expected from the characteristic fragmentations of the requisite monosubstituted benzenes.^3^ Both these *ortho* effects can be readily interpreted by mechanisms involving hydrogen transfer through a six-membered ring transition state.
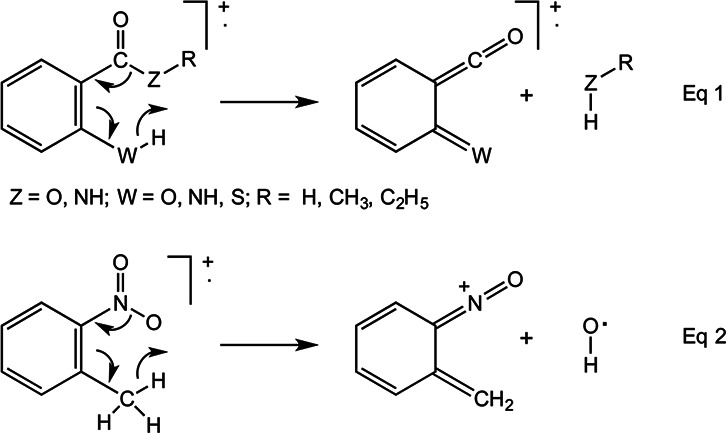


Other cases include the preferential loss of water from ionised phenols containing an *ortho* alkyl group; in this instance, the initial hydrogen transfer occurs through a five-membered ring, with eventual formation of a radical-cation that can be formulated either as a distonic ion or as ionised norcaratriene, in which a new three-membered ring is fused to the aromatic nucleus, equation (3).^4^
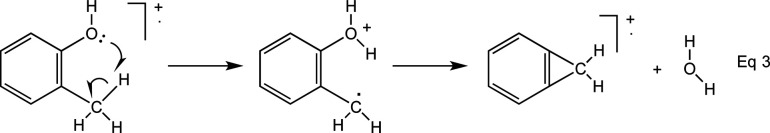


Although the signal for loss of water from ionised 2-methylphenol is not pronounced (Relative Intensity, RI ∼ 22%), it is significantly stronger than the corresponding signal in the spectra of 3-methylphenol and 4-methylphenol (RI ∼ 12% and 8%, respectively). Moreover, explusion of water competes better with loss of a hydrogen atom in the fragmentation of ionised 2-methylphenol than for either of the other two isomers.

A much more noticeable *ortho* effect, equation (4), occurs in the loss of water from the homologue, ionised 2-methylbenzyl alcohol, in which it gives rise to the base peak, which is over twice as intense as any other signal in the spectrum. The peak for [M – H_2_O]^+^ in the spectra of 3-methylbenzyl and 4-methylbenzyl alcohol is much less prominent (RI ∼ 20%) and is weaker than at least five other signals in each of those isomeric cases.^4^
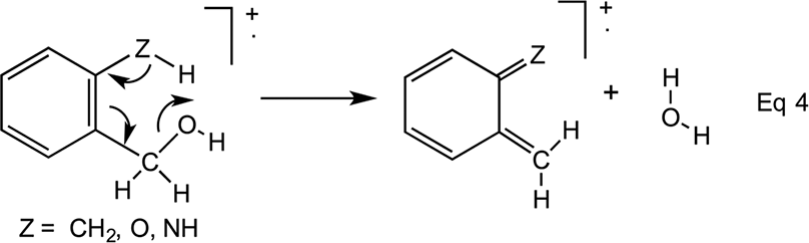


Similar trends are found in the spectra of isomeric hydroxybenzyl alcohols and aminobenzyl alcohols. In both series, the signal for [M – H_2_O]^+^ in the spectrum of the 2-isomer is far stronger and permits it to be distinguished from the 3-isomer and 4-isomer.^4^

**Scheme 1.**
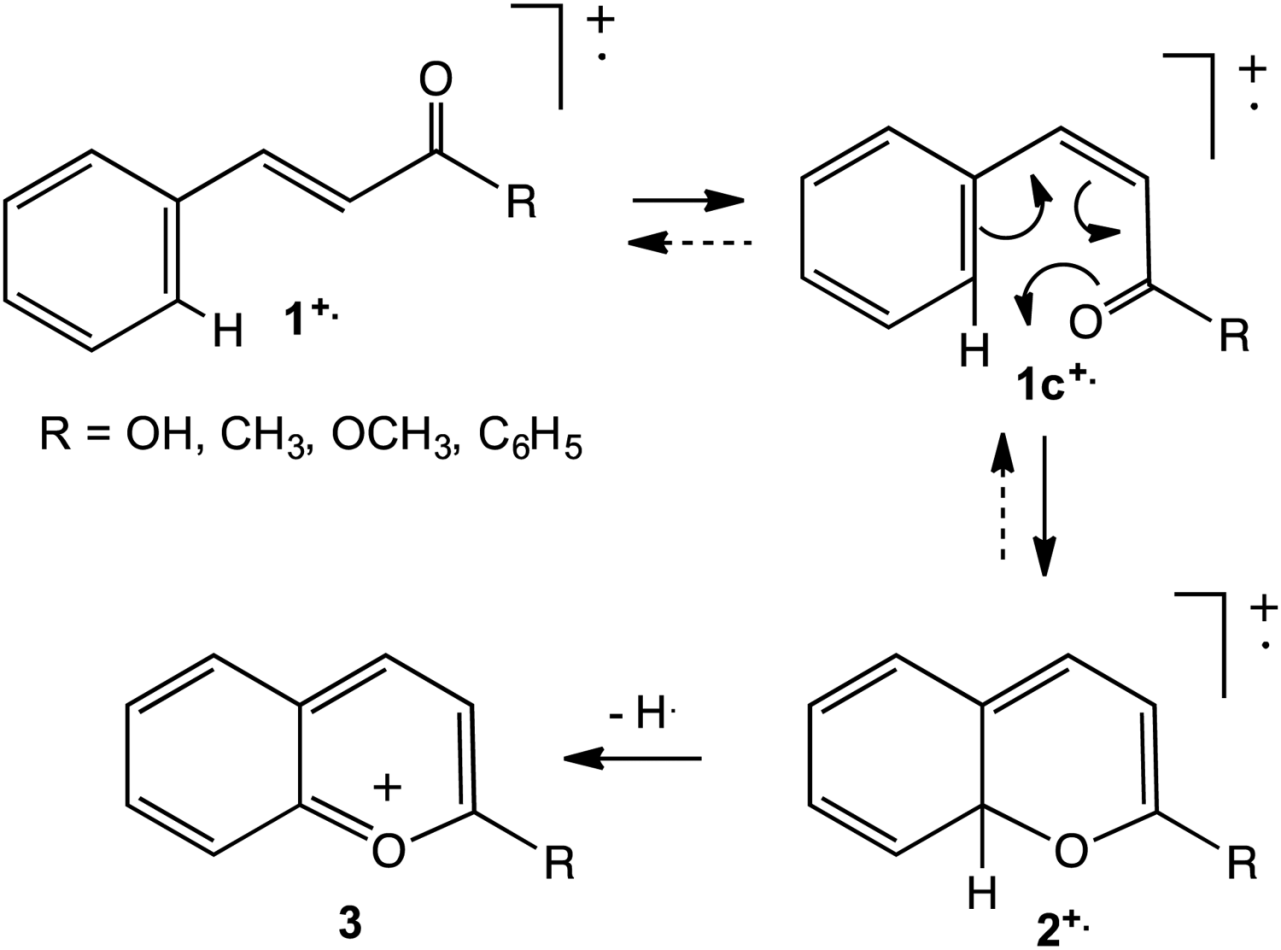
General mechanism of loss of H^·^ from C_6_H_5_CH=CHCOR^+·^

Another general class of rearrangement that may be applied to differentiate substrates containing an *ortho* substituent from their *meta* and *para* isomers was discovered from the observation that the electron ionisation (EI, historically known as electron impact) mass spectra of cinnamic acid [C_6_H_5_CH=CHCO_2_H], methyl cinnamate [C_6_H_5_CH=CHCO_2_CH_3_] and cinnamoyl ketones [C_6_H_5_CH=CHCOR, R = CH_3_ or C_6_H_5_] all contain strong signals corresponding to [M – H]^+^. This process, which was then surprising, was explained by postulating cyclisation of the *cis* geometrical isomer, **1c^+^**^·^, of the parent radical-cation, **1^+^**^·^, to form **2^+^**^·^, from which H· is lost to give the resonance-stabilised ion **3**, Scheme 1.^5^

Labelling experiments, which showed that the eliminated hydrogen atom originates from the aromatic ring, support this mechanism.^5^ Further evidence was obtained in a study that established that the 2-methylbenzopyrylium cation [**3**, R = CH_3_] is formed by loss of an *ortho* substituent from ionised benzalacetones [XC_6_H_4_CH=CHCOCH_3_]^+·^.^6^ The mechanism of these fragmentations, which may be interpreted as intramolecular aromatic substitutions, was later probed in considerable detail,^7–10^ leading to a comprehensive review.^11^ An alternative name for these processes, which emphasises their analytical significance, is ‘proximity effects’. They differ from *ortho* effects in so far as cyclisation precedes expulsion of an *ortho* substituent (as the corresponding radical or atom), with formation of an exceptionally stable cation with an extended aromatic π-system.

Studies of naturally occurring heterocyclic systems such as aurones confirmed that proximity effects have considerable analytical potential.^12,13^ This usefulness was confirmed by mass spectrometric investigations of industrially important heterocycles, including 2-styrylbenzazoles, for which prominent [M – X]^+^ signals were specifically associated with the presence of a substituent, X, in the 2-position of the pendant aromatic ring, equation (5).^14,15^ Recent work has focused on establishing more precisely the circumstances in which proximity effects compete effectively with simple cleavages.^16,17^
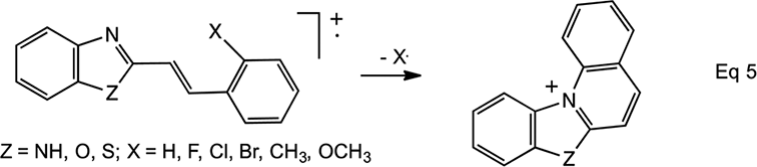


In the case of ionised substituted 2-styrylbenzimidazole (equation (5), Z = NH), the proximity effect occurs so readily that loss of X· is the dominant fragmentation when X is in the 2-position of the pendant aromatic ring, even when X = F.^14^ In contrast, significant [M – X]^+^ signals appear in the mass spectra of 2-substituted benzanilides, 2-XC_6_H_4_NHCOC_6_H_4_Y, only when X = Cl, Br, I or, to a lesser extent, CH_3_O.^16^ Nevertheless, the presence of [M – X]^+^ signals in the mass spectra of some 2-substituted benzanilides reveals that the proximity effect does occur in some cases when the initial cyclisation involves a 5-membered ring. This divergent behaviour reflects the absence of facile simple cleavages in ionised 2-styrylbenzimidazoles, whereas fission of the NH-CO bond in ionised benzanilides leads directly to the stable substituted benzoyl cation, [YC_6_H_4_CO]^+^.

Cinnamic acids and their derivatives have been applied in a variety of contexts: for example, they are important in perfumery,^18^ they have potential as anti-cancer agents^19^ and they possess antioxidant and antimicrobial properties.^20^ However, the corresponding cinnamic acid amides (cinnamamides) have received far less attention, though certain examples obtained from natural sources affect the germination and growth of lettuce, tomatoes and onions,^21^ while others show promise as α-glucosidase inhibitors.^22^ This systematic study of the EI spectra of 53 substituted cinnamamides, many of which are novel and have not previously been investigated, was initiated to explore the analytical value of proximity effects in obtaining structural information on this series of compounds.

## Experimental

### Synthesis

The substituted cinnamic acids were prepared by condensation of the corresponding aryl aldehyde with excess malonic acid in pyridine containing a catalytic quantity of piperidine.^23^ Most of the requisite aryl aldehydes were commercial samples of high purity, but some ‘mixed’ dihalogenoarylaldehydes [XYC_6_H_3_CHO; X, Y = F, Cl or Br] were prepared from the corresponding dihalogenotoluenes [XYC_6_H_3_CH_3_] by radical-initiated bromination^23^ with two equivalents of N-bromosuccinimide to give the dibromomethyl derivative [XYC_6_H_3_CHBr_2_], which was carefully hydrolysed with calcium carbonate and water;^23^ steam distillation then afforded the desired aryl aldehyde. The corresponding cinnamamides were prepared by the treatment of the parent acid with thionyl chloride; after removal of the excess thionyl chloride by distillation, the unpurified cinnamoyl chloride was added dropwise to excess aqueous ammonia solution (specific gravity 0.88).^23^ The crude cinnamamides were isolated by extraction into ethyl acetate and recrystallised either from ethyl acetate or a mixture of ethyl acetate and petroleum ether. The molecular formula of each cinnamamide was established by high resolution mass spectrometry; the structure of each compound was confirmed by mass spectrometry and ^1^H nuclear magnetic resonance (NMR) spectroscopy. Full particulars, including the melting point of each cinnamamide, many of which are novel compounds, are given in the supplementary information. No impurities in the recrystallised cinnamamides were detected by NMR or mass spectrometry.

### Mass spectrometry

The mass spectra were acquired by means of a 7890 gas chromatograph attached to a 5975 Electron Ionization Inert MSD (Agilent Technologies, USA) instrument. Gas chromatography (GC) was achieved with a 30 m × 0.25 mm 5% diphenyl low-polarity fused-silica capillary column, using helium as the carrier gas at a flow rate of 1.2 mL/min. Ionisation was effected with electrons having a nominal energy of 70 eV. The temperature of the source and quadrupole was 230 °C and 150 °C, respectively. The initial temperature of the GC was 100 °C, increasing linearly at 25 °C/min to 350 °C, where it was maintained for 2 min. Data were acquired over the *m/z* range 50 to 600. No impurities were detected by gas chromatography mass spectrometry (GCMS), thus confirming that the cinnamamides were pure and not adversely affected by the high temperatures in the instrumentation.

## Results and discussion

The EI mass spectra of the cinnamamides are summarised in Tables 1–7. In order to facilitate the discussion, a system of abbreviation is used to describe the substitution pattern of the cinnamamides: the stem ‘CAm’ denotes cinnamamide; the prefix delineates the nature and position of the substituent. Thus, ‘2Br4ClCAm’ denotes ‘2-bromo-4-chlorocinnamamide’.

**Table 1.** Summary of electron ionisation mass spectra of XC_6_H_4_CH=CHCONH_2_.

X																		
H		F				Cl				Br				I				Interpretation^a^
*m/z*	*RI* ^b^	*m/z*	*RI* ^b^			*m/z*	*RI* ^b^			*m/z*	*RI* ^b^			*m/z*	*RI* ^b^			
			2^c^	3^c^	4^c^		2^c^	3^c^	4^c^		2^c^	3^c^	4^c^		2^c^	3^c^	4^c^	
						184	∼0	2	2	228	∼0	5.6	5.5					^13^C sat of [M + 2]^+.^
						183	2.9	20	21	227	5	57	57					[M + 2]^+.^
148	5	166	8	6	7	182	1.1	38^d^	39^d^	226	∼0	100^e^	100^e^	274	∼0	7.7	9.4	^13^C sat of M^+.^
147	47^f^	165	81^f^	64^f^	67^f^	181	8.9	62^f^	64^f^	225	6	58^f^	58^f^	273	∼0	77^f^	95^f^	M^+.^
146	100	164	42	100	100	180	∼0	100	100	224	∼0	98	97	272	∼0	100	100	[M – H]^+^
131	45	149	91	70	79	165	4.8	63	74	209	2.5	45	53	257	∼0	14	21	[M – NH_2_]^+^
[146]		146	68	6	7	146	100	7	3.6	146	100	11	6	146	100	9	7.5	[M – X]^+^
103	58	121	77	51	60	137	11	36	40	181	3.9	21	22	229	∼0	1.3	3.8	[M – NH_2_ – CO]^+^
77	35	95	8	10	11	111	∼0	4	4.4	155	∼0	2.5	2.2	203	∼0	∼0	∼0	[M – NH_2_ – CO – C_2_H_2_]^+^
102	16	102	10	6	6	102	12	45	42	102	26	100	100	102	23	48	55	[M – NH_2_ – CO – X]^+.^
		101	100	58	61	101	20	36	42	101	12	21	22	101	5.8	8.6	10	[M – NH_2_ – CO – HX]^+^

^a^ The data are arranged so that the *m/z* values in each row correspond to a common interpretation; with the exception of signals in the molecular ion region, ^13^C, ^37^Cl and ^81^Br isotope satellites are not included.

^b^ *RI* measured by peak height and normalised to a value of 100 units for the base peak.

^c^ The number at the head of these columns indicates the position of X in the aromatic ring.

^d^ Most of this signal is the ^37^Cl satellite of [M – H]^+^.

^e^ Most of this signal is the ^81^Br satellite of [M – H]^+^.

^f^ Part of this signal is the ^13^C satellite of [M – H]^+^.

**Table 2.** Summary of electron ionisation mass spectra of XC_6_H_4_CH=CHCONH_2_.

X																
CH_3_				CH_3_O				CF_3_				NO_2_				Interpretation^a^
*m/z*	*RI* ^b^			*m/z*	*RI* ^b^			*m/z*	*RI* ^b^			*m/z*	*RI* ^b^			
	2^c^	3^c^	4^c^		2^c^	3^c^	4^c^		2^c^	3^c^	4^c^		2^c^	3^c^	4^c^	
162	5.1	6.8	7.1	178	1.3	9.4	11	216	2.5	6.7	5.4	193	∼0	6.1	6.3	^13^C sat of M^+.^
161	44	63^d^	66^d^	177	11	83^d^	100^d^	215	22	62^d^	51^d^	192	∼0	54^d^	55^d^	M^+.^
160	17	100	100	176	1.6	100	67	214	4.5	100	100	191	∼0	35	100	[M – H]^+^
145	24	41	49	161	7	43	63	199	16	89	64	176	1.0	86	59	[M – NH_2_]^+^
146	76	37	17	146	100	12	3.6	146	93	4.8	5.5	146	100	20	12	[M – X]^+^
117	69	30	32	133	2.8	19	31	171	9	56	47	148	14	12	1.8	[M – NH_2_ – CO]^+^
91	31	23	21	107	∼0	∼0	∼0	145	2	8.9	7.7	122	∼0	∼0	∼0	[M – NH_2_ – CO – C_2_H_2_]^+^
115^e^	100	57	54					151^e^	100	82	65	102^e^	28	100	39	

^a^ The data are arranged so that the *m/z* values in each row correspond to a common interpretation.

^b^ *RI* measured by peak height and normalised to a value of 100 units for the base peak.

^c^ The number at the head of these columns indicates the position of X in the aromatic ring.

^d^ Part of this signal is the ^13^C satellite of [M – H]^+^.

^e^ Data are included to define the base peak in certain spectra.

**Table 3.** Summary of electron ionisation mass spectra of Cl_2_C_6_H_3_CH=CHCONH_2_.

	*RI* ^a^						
*m/z*	2,3^b^	2,4^b^	2,5^b^	2,6^b^	3,4^b^	3,5^b^	Interpretation^c^
219	1.2	<1	1.3	<1	8.4	7.6	^37^Cl_2_ sat of M^+.^
218	∼0	∼0	∼0	∼0	15^d^	15^d^	^37^Cl_2_ sat of [M – H]^+^
217	7.3	4.0	7.7	5.1	50	46	^37^Cl sat of M^+.^
216	1.6	∼0	1.5	∼0	72^e^	71^e^	^37^Cl sat of [M – H]^+^
215	11	6.1	12	7.8	76^f^	71^f^	M^+.^
214	∼0	∼0	∼0	∼0	100	100	[M – H]^+^
199	5.1	4.2	5.2	4.4	86	78	[M – NH_2_]^+^
180	100	100	100	100	7.2	12	[M – Cl]^+^
171	10	8.6	8.3	9.3	40	30	[M – NH_2_ – CO]^+^
145	1.8	1.5	1.5	1.4	2.8	2.6	[M – NH_2_ – CO – C_2_H_2_]^+^

^a^ *RI* measured by peak height and normalised to a value of 100 units for the base peak.

^b^ The numbers at the head of these columns indicate the positions of Cl in the aromatic ring.

^c^ The data are arranged so that the *m/z* values in each row correspond to a common interpretation; with the exception of signals in the molecular ion region, ^13^C and ^37^Cl isotope satellites are not included.

^d^ Part of this signal is the ^13^C^37^Cl satellite of M^+.^.

^e^ Part of this signal is the ^13^C satellite of M^+.^.

^f^ Part of this signal is the ^13^C satellite of [M – H]^+^.

**Table 4.** Summary of electron ionisation mass spectra of ClFC_6_H_3_CH=CHCONH_2_.

	*RI* ^a^						
*m/z*	2Cl4F^b^	4Cl2F^b^	2Cl5F^b^	5Cl2F^b^	2Cl6F^b^	3Cl5F^b^	Interpretation^c^
202	∼0	2.0	∼0	3.1	∼0	2.8	^13^C^37^Cl sat of M^+.^
201	1.8	20	4.5	30	3.1	26	^37^Cl sat of M^+.^
200	∼0	13^d^	1.5	15^d^	1.0	42^d^	^37^Cl sat of [M – H]^+^
199	5.4	59^e^	14	92^e^	9.2	81^e^	M^+.^
198	∼0	20	∼0	18	∼0	100	[M – H]^+^
183	4.3	100	6.5	100	8.1	92	[M – NH_2_]^+^
180	∼0	56	∼0	56	∼0	6.3	[M – F]^+^
164	100	40	100	19	100	9.3	[M – Cl]^+^
155	12	62	13	79	15	45	[M – NH_2_ – CO]^+^
129	∼0	2.0	∼0	3.1	∼0	3.7	[M – NH_2_ – CO – C_2_H_2_]^+^

^a^ RI measured by peak height and normalised to a value of 100 units for the base peak.

^b^ The numbers at the head of these columns indicate the positions of Cl and F in the aromatic ring.

^c^ The data are arranged so that the *m/z* values in each row correspond to a common interpretation; with the exception of signals in the molecular ion region, ^13^C and ^37^Cl isotope satellites are not included.

^d^ Part of this signal is the ^13^C satellite of M^+.^.

^e^ Part of this signal is the ^13^C satellite of [M – H]^+^.

**Table 5.** Summary of electron ionisation mass spectra of BrFC_6_H_3_CH=CHCONH_2_.

	*RI* ^a^					
*m/z*	2Br4F^b^	4Br2F^b^	2Br5F^b^	5Br2F^b^	3Br5F^b^	Interpretation^c^
246	∼0	5.6	∼0	5.8	4.7	^13^C^81^Br sat of M^+.^
245	3.8	54	5.8	58	48	^81^Br sat of M^+.^
244	∼0	24	∼0	17	69^d^	^81^Br sat of [M – H]^+^
243	3.9	55^e^	6.0	59^e^	49^e^	M^+.^
242	∼0	19	∼0	12	66	[M – H]^+^
227	2.7	73	2.9	52	42	[M – NH_2_]^+^
224	∼0	52	∼0	34	2.6	[M – F]^+^
199	3.5	40	3.2	35	16	[M – NH_2_ – CO]^+^
164	100	43	100	14	8.5	[M – Br]^+^
173	∼0	∼0	∼0	∼0	1.0	[M – NH_2_ – CO – C_2_H_2_]^+^
120	31	100	34	100	100	[M – NH_2_ – CO – Br]^+.^

^a^ *RI* measured by peak height and normalised to a value of 100 units for the base peak.

^b^ The numbers at the head of these columns indicate the positions of Br and F in the aromatic ring.

^c^ The data are arranged so that the *m/z* values in each row correspond to a common interpretation; with the exception of signals in the molecular ion region, ^13^C and ^81^Br isotope satellites are not included.

^d^ Part of this signal is the ^13^C satellite of M^+.^.

^e^ Part of this signal is the ^13^C satellite of [M – H]^+^.

**Table 6.** Summary of electron ionisation mass spectra of BrClC_6_H_3_CH=CHCONH_2_.

	*RI* ^a^						
*m/z*	2Br4Cl^b^	4Br2Cl^b^	2Br5Cl^b^	5Br2Cl^b^	2Br6Cl^b^	3Br5Cl^b^	Interpretation^c^
264	∼0	∼0	∼0	∼0	∼0	1.5	^13^C^37^Cl^81^Br sat of M^+.^
263	1.3	2.3	2.2	4.3	1.3	16	^81^Br^37^Cl sat of M^+.^
262	∼0	1.1^d^	∼0	1.8^d^	∼0	29^d^	^81^Br^37^Cl sat of [M – H]^+^
261	5.4	9.4	8.7	16.8	5.2	64^e^	^81^Br or ^37^Cl sat of M^+.^
260	∼0	1.2^f^	∼0	1.6^f^	∼0	100^g^	^81^Br or ^37^Cl sat of [M – H]^+^
259	4.2	7.3	6.7	13.0	4.0	49^h^	M^+.^
258	∼0	∼0	∼0	∼0	∼0	75	[M – H]^+^
243	2.1	3.4	2.5	4.5	3.9	40	[M – NH_2_]^+^
224	∼0	100	∼0	100	58	3.7	[M – Cl]^+^
215	2.2	6.1	2.6	5.6	6.5	13	[M – NH_2_ – CO]^+^
180	100	1.7	100	1.5	100	10	[M – Br]^+^ or [M – NH_2_ – CO – Cl]^+^
136	23	23	24	26	31	79	[M – NH_2_ – CO – Br]^+.^

a *RI* measured by peak height and normalised to a value of 100 units for the base peak.

b The numbers at the head of these columns indicate the positions of Br and Cl in the aromatic ring.

c The data are arranged so that the *m/z* values in each row correspond to a common interpretation; with the exception of signals in the molecular ion region, ^13^C, ^37^Cl and ^81^Br isotope satellites are not included.

d Part of this signal is the ^13^C^81^Br or ^13^C^37^Cl satellite of M^+.^.

e Part of this signal is the ^13^C^81^Br or ^13^C^37^Cl satellite of [M – H]^+^.

f Most of this signal is the ^13^C satellite of M^+.^.

g Part of this signal is the ^13^C satellite of M^+.^.

h Part of this signal is the ^13^C satellite of [M – H]^+^.

**Table 7.** Summary of electron ionisation mass spectra of 3,4(CH_3_O)_2_C_6_H_3_CH=CHCONH_2_, 3,4(OCH_2_O)C_6_H_3_CH=CHCONH_2_ and 4(CH_3_)*_n_*CH*_3−n_*C_6_H_4_CH=CHCONH_2_.

3,4X_2_C_6_H_3_CH=CHCONH_2_^a^				4(CH_3_)*_n_*CH*_3−n_*C_6_H_4_CH=CHCONH_2_^a,b^						
3,4(CH_3_O)_2_^a^		3,4(OCH_2_O)^a^		4CH_3_CH_2_ (*n* = 1)^a,b^		4(CH_3_)_2_CH (*n* = 2)^a,b^		4(CH_3_)_3_C (*n* = 3)^a,b^		
*m/z*	*RI* ^c^	*m/z*	*RI* ^c^	*m/z*	*RI* ^c^	*m/z*	*RI* ^c^	*m/z*	*RI* ^c^	Interpretation^d^
208	13	192	12	176	8.0	190	10	204	4.5	^13^C sat of M^+.^
207	100^e^	191	100^e^	175	67^e^	189	78^e^	203	31^e^	M^+.^
206	40	190	54	174	100	188	66	202	6.6	[M – H]^+^
192	14^f^	176	2.6^g^	160	13^f^	174	100^f^	188	100	[M – CH_3_]^+^
191	22	175	23	159	29	173	6.6	187	∼0	[M – NH_2_]^+^
163	6.0	147	6.8							[M – NH_2_ – CO]^+^

a The numbers at the head of these columns indicate the positions of the substituents in the aromatic ring.

b The number, *n*, in these formulae indicates the number of methyl groups in the 4-alkyl substituent.

c *RI* measured by peak height and normalised to a value of 100 units for the base peak.

d The data are arranged so that the *m/z* values in each row correspond to a common interpretation; with the exception of signals in the molecular ion region, ^13^C isotope satellites are not included.

e Part of this signal is the ^13^C satellite of [M – H]^+^.

f Part of this signal is the ^13^C satellite of [M – NH_2_]^+^.

g Almost all of this signal is the ^13^C satellite of [M – NH_2_]^+^.

Tables 1 and 2 summarise the spectra of the parent compound and a wide range of monosubstituted cinnamamides, XC_6_H_4_CH=CHCONH_2_. Several salient points immediately emerge from these data.

Firstly, the proximity effect operates consistently in the fragmentation of each of these ionised cinnamamides. The base peak in the spectrum of the parent, CAm, corresponds to [M – H]^+^ (relative intensity, RI, almost twice that of the next most intense signal). Similarly, abundant [M – X]^+^ ions are found in the spectra of all 2-XCAms (relative abundance, RA, in the range 68%, for X = F, to 100%, for X = Cl, Br, I, CH_3_O or NO_2_). In these cases, the RA of [M – H]^+^ declines dramatically (to a maximum of 42% for X = F, through 17% for X = CH_3_, 4.5% for X = CF_3_, 1.6% for X = CH_3_O, to negligible importance for X = Cl, Br, I or NO_2_). These results reveal that the proximity effect occurs more readily for a greater variety of substituents for ionised cinnamamides than for the analogous ionised benzanilides, for which [M – X]^+^ signals are significant only when X = Cl, Br, I or, to a lesser extent, CH_3_O.^16^

**Scheme 2.**
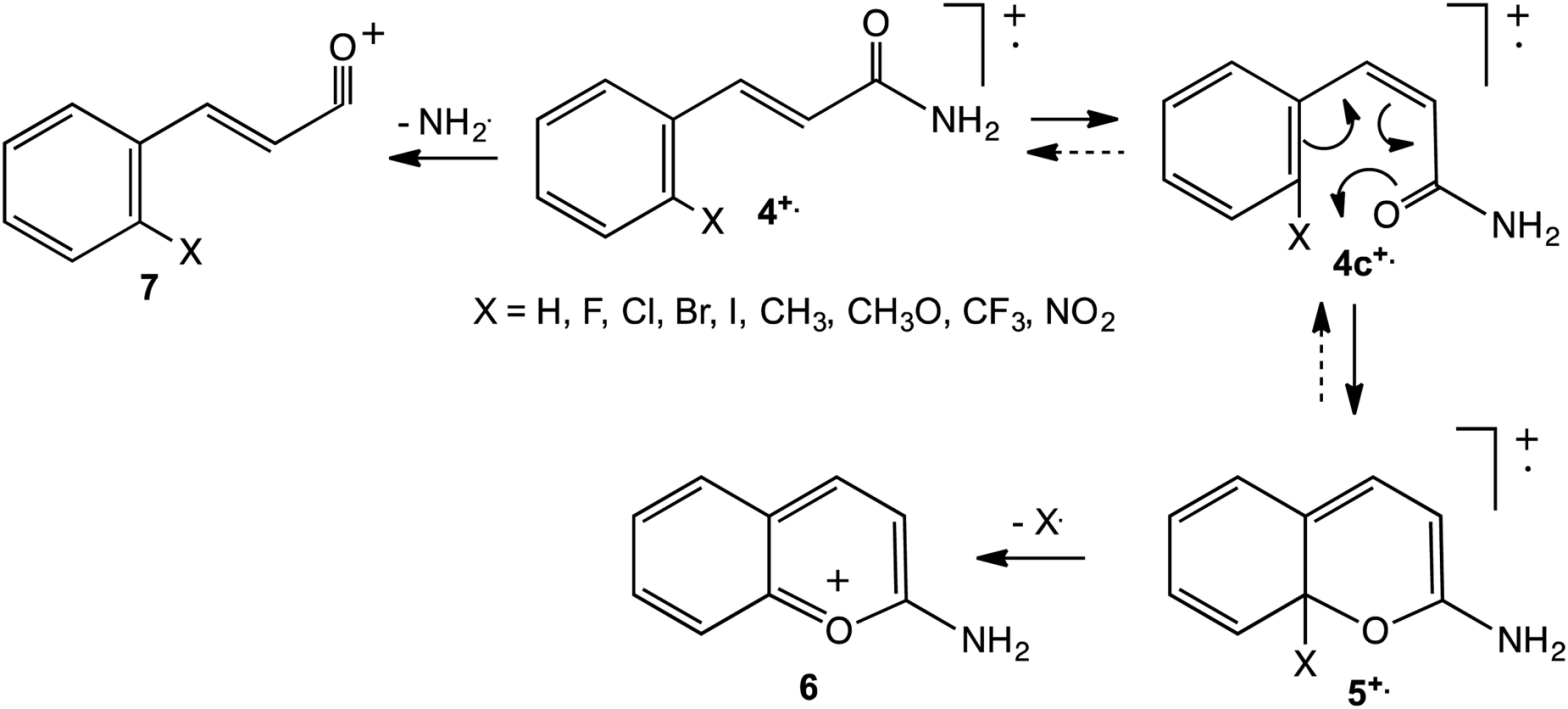
Mechanism for loss of X^·^ from 2-XC_6_H_4_CH=CHCONH_2_^+·^

Secondly, there is an excellent general correlation between the formation of strong signals for [M – X]^+^ and the presence of the substituent, X, in the 2-position. However, when X is in the 3-position or 4-position, signals for [M – H]^+^ are usually far stronger than peaks corresponding to [M – X]^+^. Thus, when X = F, the ratio of the RA of [M – X]^+^ to that of [M – H]^+^ is 1.6:1 when X is in the 2-position, but only 0.06:1 and 0.07:1, respectively, when it is in the 3-position or 4-position. The corresponding ratios when X = CH_3_ in the 2-position, 3-position and 4-position are 4.5:1, 0.37:1 and 0.17:1, respectively. Even more spectacular variations are found when X = CF_3_ (21:1, 0.05:1 and 0.05:1) or CH_3_O (63:1, 0.12:1 and 0.05:1) and when X = Cl, Br, I or NO_2_, for which the ratio is at least 500:1 when X is in the 2-position, but in the range 0.03:1 to 0.57:1 when it is in the 3-position or 4-position. Scheme 2 accounts for these observations: isomerisation of **4^+^**^·^ to its *cis* isomer, **4c^+^**^·^, allows cyclisation to **5^+^**^·^, from which X^·^ may be expelled to form the extremely stable 2-aminobenzopyrylium cation, **6**. An amino substituent is exceptionally effective at stabilising a cation by π-conjugation; moreover, it is ideally located in the 2-position to stabilise the benzopyrylium cation. When X is in the 3-position or 4-position, loss of H^·^ occurs instead by the general mechanism depicted in Scheme 1 (R = NH_2_).

Thirdly, formation of [M – X]^+^ by the proximity effect occurs progressively more readily in the fragmentation of the ionised 2-XCAms on proceeding from F, through Cl and Br, to I. This trend follows that found for the corresponding 2-XC_6_H_4_NHCOC_6_H_4_Y^+·^ radical-cations,^16^ but abundant [M – X]^+^ ions are found even when X = F. The proximity effect is, therefore, more facile for the ionised cinnamamides than for the corresponding ionised benzanilides, thus enhancing its analytical value.

Fourthly, the only simple cleavage that competes effectively with the proximity effect is loss of an amino radical to form a cinnamoyl cation, [XC_6_H_4_CH=CHCO]^+^, Scheme 2. With the exception of the series when X = F, the RA of [M – NH_2_]^+^ signal is always higher when X is in the 3-position or 4-position as opposed to the 2-position. This trend emphasises how the proximity effect generally competes more effectively with simple cleavages when X is in the 2-position. In certain cases, signals that may be assigned to secondary and tertiary fragment ions corresponding to [M – NH_2_ – CO]^+^ and [M – NH_2_ – CO – C_2_H_2_]^+^ are significant, especially when X = F, CH_3_ and CF_3_. The importance of these peaks, particularly those that are attributable to tertiary fragmentation of the molecular ion, M^+·^, declines rapidly on progressing from X = F through Cl and Br to I, even for members of the 3CAm and 4CAm series.

**Figure 1.**
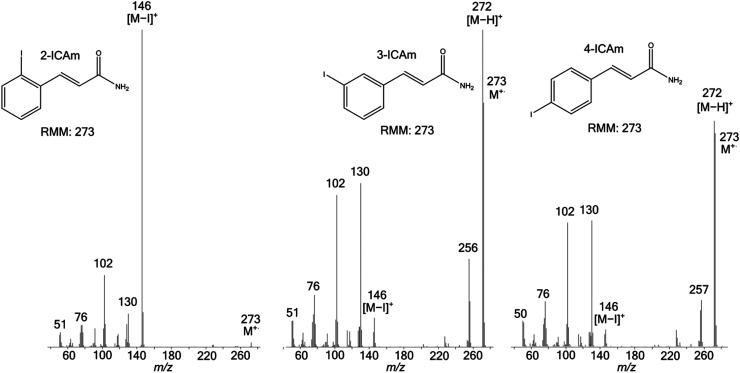
Electron ionisation mass spectra of 2ICAm (left), 3ICAm (middle) and 4ICAm (right).

These trends are well-illustrated by the spectra of the three isomeric ICAms, Figure 1, for which cleavage of the relatively weak C-I bond would be expected to compete most effectively with the proximity effect. However, the RI of the signal for [M – I]^+^ in the spectra of 3ICAm and 4ICAm is only 9% and 7.5%, respectively, whereas [M – H]^+^ formed by the proximity effect gives rise to the base peak. In contrast, the spectrum of 2ICAm contains only very weak signals corresponding to M^+.^ and [M – H]^+^, but is instead dominated by the peak for [M – I]^+^. Moreover, the RIs of the peaks corresponding to [M – NH_2_]^+^ and [M – NH_2_ – CO]^+^ in the spectra of 3ICAm and 4ICAm lie in the range 14% to 21% and 1.3% to 3.8%, respectively, but these signals are negligibly weak in the spectrum of 2ICAm.

These and related trends indicate that as the strength of the C-X bond declines on progressing from F through Cl and Br to I, ions arising by cleavage of this bond become more important, but they are always much less abundant than the [M – X]^+^ ions formed by the proximity effect, especially when X is in the 2-position.

Table 3 summarises the EI spectra of the six isomeric Cl_2_CAms. These data reinforce and augment the deductions made from the spectra of isomeric XCAms.

Firstly, despite the complications caused by the ^37^Cl_2_ and ^37^Cl isotope satellites in the molecular ion region, the base peak in the spectra of both 3,4Cl_2_CAm and 3,5Cl_2_CAm corresponds to [M – H]^+^, even though two chloro substituents are present in the 3-position, 4-position or 5-position. The peaks corresponding to [M – H]^+^ in the spectra of the other four isomers (2,3CAm, 2,4CAm, 2,5CAm and 2,6CAm) are too weak to be measured reliably. This trend confirms that loss of a hydrogen atom by the proximity effect occurs with high selectivity only if there is no relatively weak C-X bond in the 2-position that can be cleaved in the last step of the proximity effect to form [M – X]^+^.

Secondly, the spectra of all four isomers with one or two chloro substituent(s) in the *ortho* position relative to the CH=CHCONH_2_ side chain are dominated by the signal corresponding to [M – Cl]^+^, which typically has an RI more than 10 times that of any other peak. In contrast, the spectra of 3,4Cl_2_CAm and 3,5Cl_2_CAm contain far weaker signals for [M – Cl]^+^ (RI = 7.2% and 12%, respectively), again illustrating the analytical value of the proximity effect.

Thirdly, the RI of the signal corresponding to M^+·^ is higher in the spectra of both 3,4Cl_2_CAm and 3,5Cl_2_CAm (76% and 71%, respectively, though part of this signal corresponds to the ^13^C satellite of [M – H]^+^), than in the spectra of the other four isomers (RI = 6–12%). This trend establishes two valuable general correlations. The RI of the peak for M^+·^ is much higher when there is no chloro substituent in the 2-position; loss of a chlorine atom from the 2-position by the proximity effect is so favourable that it leads to a profound decline in the RA of M^+·^. In addition, in cases in which either a chlorine or a hydrogen atom may be lost by the proximity effect, formation of [M – Cl]^+^ pre-empts production of [M – H]^+^ almost entirely.

Fourthly, simple cleavage to yield [M – NH_2_]^+^ competes much more effectively with the proximity effect when there is no 2-chloro substituent. The RI of the peak produced by this simple cleavage is 86% and 78%, respectively, in the spectra of 3,4CAm and 3,5CAm, but only 4.2% to 5.2% in the spectra of the other four isomeric ionised Cl_2_CAms, for which loss of a chlorine atom by the proximity effect occurs preferentially.

**Figure 2.**
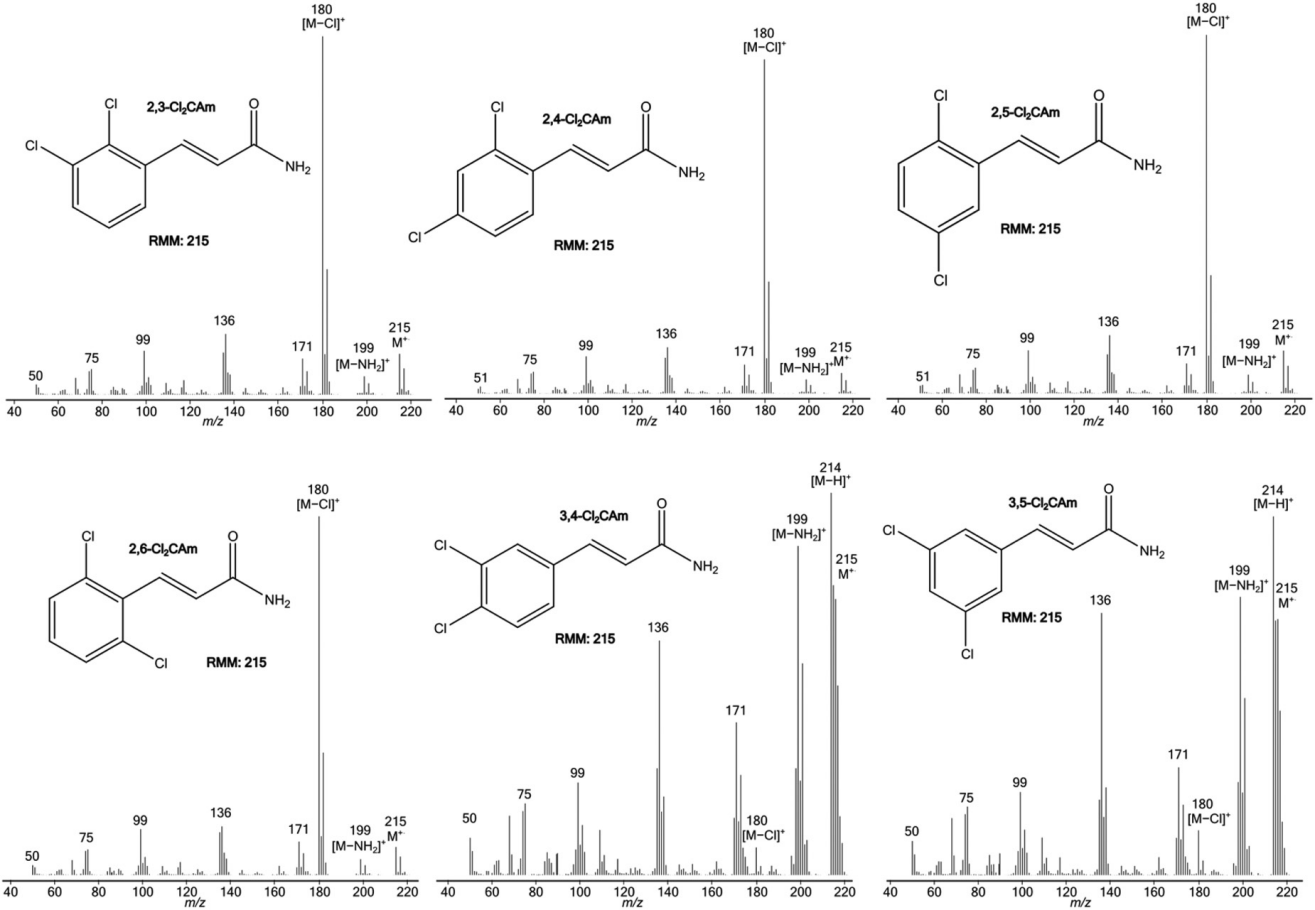
Electron ionisation mass spectra of 2,3Cl_2_CAm (top left), 2,4Cl_2_CAm (top middle), 2,5Cl_2_CAm (top right), 2,6Cl_2_CAm (bottom left), 3,4Cl_2_CAm (bottom middle) and 3,5Cl_2_CAm (bottom right).

The spectra of the six isomeric Cl_2_CAms shown in Figure 2 underline the analytical value of the proximity effect.

The spectra of isomeric pairs of ‘mixed’ dihalogenocinnamamides, XYC_6_H_3_CH=CHCONH_2_ are summarised in Tables 4–6. These data shed further light on the circumstances in which a halogen atom is lost from ionised CAms by the proximity effect.

Firstly, significant signals corresponding to [M – H]^+^ appear in the spectra of the XYCAms only if there is neither a 2-bromo nor a 2-chloro substituent. This observation confirms that loss of a hydrogen atom by the proximity effect is effectively pre-empted by expulsion of a bromine or chlorine atom from the 2-position to produce, respectively, [M – Br]^+^ and [M – Cl]^+^ ions that invariably give rise to the base peak in the spectra. Moreover, even in those cases in which there is a 2-fluoro substituent, the peak corresponding to [M – F]^+^ is typically two or three times as intense as that for [M – H]^+^. The spectrum of 3Br5ClCAm contains a strong signal (RI = 75%) for [M – H]^+^; in contrast, the spectra of all the other five BrClCAms show negligibly weak peaks (RI < 0.5%) corresponding to loss of a hydrogen atom. These results confirm that loss of a halogen atom of any kind from the 2-position is much more favourable than loss of a hydrogen atom.

Secondly, loss of a halogen atom is strongly associated with the presence of a substituent in the 2- (or 6-) position. Thus, the spectrum of 3Cl5FCAm shows only weak signals for [M – Cl]^+^ and [M – F]^+^ (RI = 9.3% and 6.3%, respectively). Similarly, the spectra of 3Br5FCAm and 3Br5ClCAm contain only weak peaks (RI = 2–10%) corresponding to [M – Br]^+^, [M – Cl]^+^ and [M – F]^+^. These data confirm the strong correlation between loss of a halogen atom and the presence of a 2-halogeno substituent. The loss of the heavier halogen atom from the 3-position always occurs more readily than expulsion of the lighter halogen atom from the 5-position, as would be expected because the strength of the C-X bond is greatest when X = F. These data confirm that loss of a halogen atom from the 3-position or 5-position by simple cleavage is of only minor significance, even when X = Br (as was observed for 3ICAm, Table 1 and Figure 1).

Thirdly, the spectra of isomeric pairs of general structure 2X4YCAm or 4X2YCAm and 2X5YCAm or 5X2YCAm, Tables 4–6, are especially informative. In every case, loss of X^·^ or Y^·^ from the 2-position occurs more readily than expulsion of Y^·^ or X^·^ from the 4-position or 5-position. This trend applies even in the preferential formation of [M – F]^+^ over either [M – Cl]^+^ or [M – Br]^+^, but the loss of the heavier halogen atom does compete to a limited extent in these cases. Even stronger trends are observed in the spectra of CAms in which there is a 2-chloro or 2-bromo substituent: the base peak always corresponds to loss of a halogen atom from the 2-position, regardless of the position of the second halogeno substituent. Indeed, in cases where there is a fluorine atom in the 4-position or 5-position, formation of [M – F]^+^ is effectively pre-empted by production of [M – Cl]^+^ or [M – Br]^+^ by expulsion of a chlorine or bromine atom from the 2-position. Similar trends are found in the spectra of 2Br4ClCAm and 2Br5ClCAm, both of which have the signal for [M – Br]^+^ as the base peak with a negligibly weak signal (RI <0.5%) for [M – Cl]^+^. In contrast, the corresponding spectra of 4Br2ClCAm and 5Br2ClCAm are dominated by signals corresponding to [M – Cl]^+^ (as the base peak), with very weak signals for [M – Br]^+^ (RI = 1–2%). All these data underline the analytical utility of the proximity effect in providing information on the position of any halogeno substituent(s) in the CAms.

The preference for eliminating a halogen atom from the 2-position appears to be even more pronounced for ionised 2X5YCAms than for the isomeric 2X4YCAms. The effect of the halogeno substituent on the intermediates corresponding to **2^+^**^·^ (in Scheme 1) or **5^+^**^·^ (in Scheme 2) may explain the enhanced preference shown by the ionised 2X5YCAms for undergoing the proximity effect. The carbocyclic ring of these intermediates contains a structural entity that resembles a pentadienyl cation, in which the lowest unoccupied molecular orbital (LUMO) has lobes at positions 1, 3, and 5, but nodes at positions 2 and 4.^24^ Positions 2 and 3 of the pentadienyl entity in the intermediate **2^+^**^·^ and **5^+^**^·^ correspond, respectively, to positions 4 and 5 in the CAm. Consequently, electron donation from a halogeno substituent in the intermediate derived from 2X5YCAm^+·^ will be more effective because there is a large lobe in the LUMO of the pentadienyl cation. In contrast, the analogous electron donation in the intermediate derived from 2X4YCAm^+·^ is unfavourable because the halogeno substituent is attached to a carbon atom at which there is a node in the LUMO.

**Figure 3.**
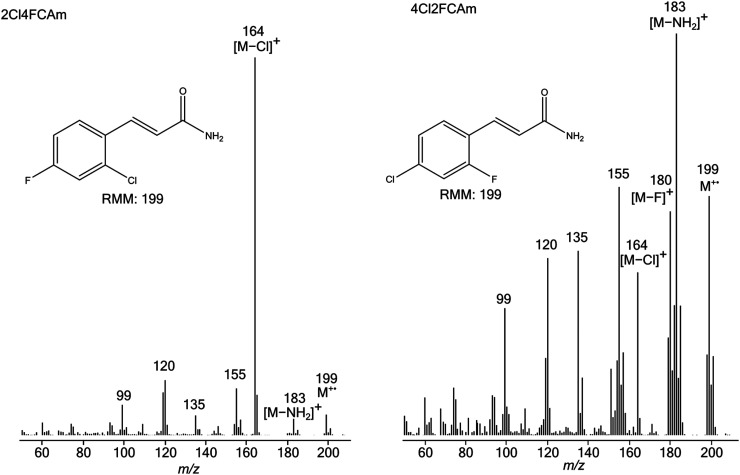
Electron ionisation mass spectra of 2Cl4FCAm (left) and 4Cl2FCAm (right).

**Figure 4.**
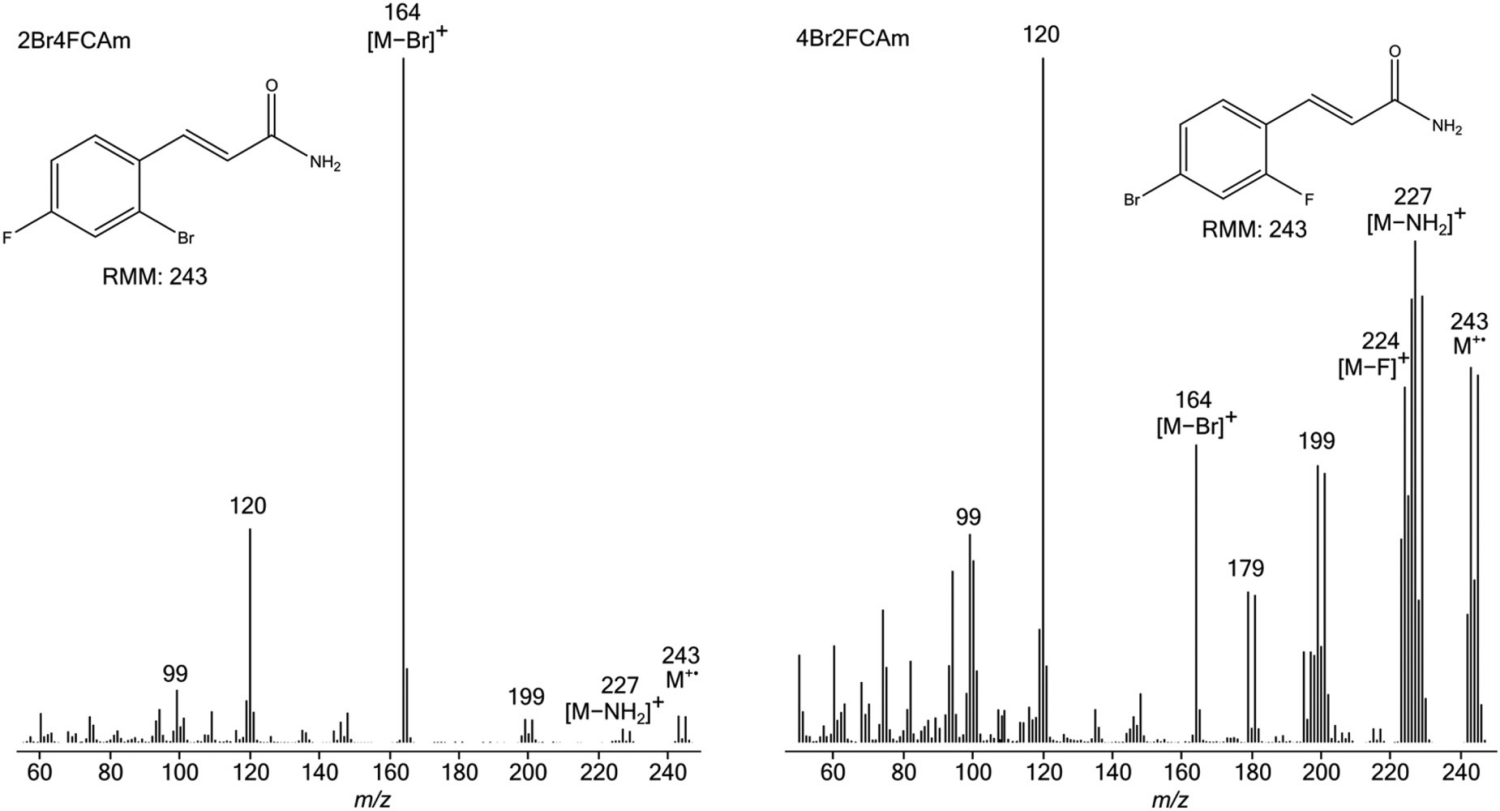
Electron ionisation mass spectra of 2Br4FCAm (left) and 4Br2FCAm (right).

**Figure 5.**
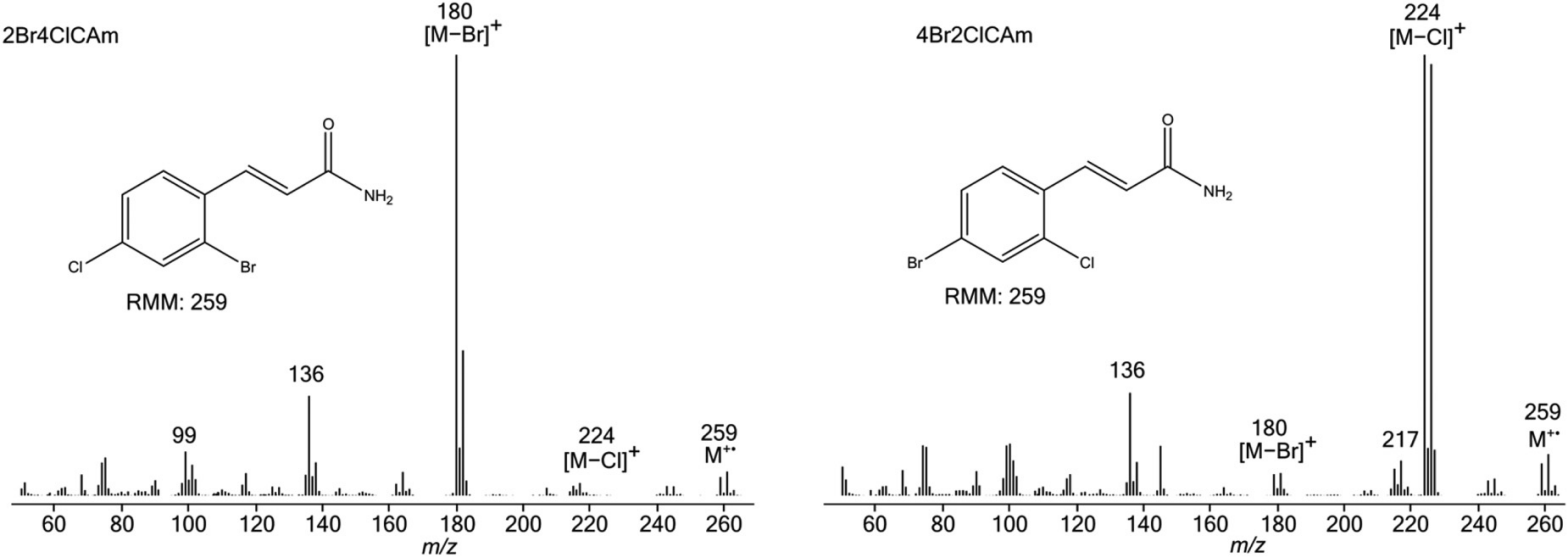
Electron ionisation mass spectra of 2Br4ClCAm (left) and 4Br2ClCAm (right).

Although this trend could in principle be applied to differentiate isomeric pairs of 2X4YCAms and 2X5YCAms, it would be unwise to place too great a reliance on this secondary feature of the spectra unless both were available and had been recorded under identical conditions. The distinctive differences of the spectra of isomeric pairs of 2X4YCAms are obvious from the representative examples of Figures 3–5.

Fourthly, when there is a 2-halogeno and a 6-halogeno substituent, loss of the heavier halogen atom by the proximity effect occurs preferentially. This effect is extremely pronounced in the spectrum of 2Cl6FCAm, in which the base peak corresponds to [M – Cl]^+^ but the signal for [M – F]^+^ is negligibly weak (RI < 0.5%). The spectrum of 2Br6ClCAm is complicated because the [M – Br]^+^ and [M – NH_2_ – CO – Cl]^+^ ions are isobaric. However, the very weak (RI < 2%) peak corresponding to [M – NH_2_ – CO – Cl]^+^ in the spectra of 4Br2ClCAm and 5Br2ClCAm suggests that practically all the base peak at *m/z* 180 in the spectrum of 2Br6ClCAm corresponds to [M – Br]^+^; the RA of [M – Cl]^+^ is only 58%. These results confirm that loss of a halogen atom by the proximity effect occurs more readily as the mass of the halogeno substituent increases and the strength of the C-X bond declines; moreover, ejection of a fluorine atom is much less favourable than expulsion of either a chlorine or bromine atom. Similar trends have been found in other systems.^14–16^

Fifthly, valuable confirmatory information can be obtained from the RI of signals corresponding to [M – NH_2_]^+^ and [M – NH_2_ – CO]^+^ ions formed by simple cleavage. This fragmentation pattern competes effectively with the proximity effect only when there is neither a 2-Br nor a 2-Cl substituent. Thus, [M – NH_2_]^+^ gives rise to the base peak in the spectra of 4Cl2FCAm and 5Cl2FCAm; the corresponding signal has an RI of 92% in the spectrum of 3Cl5FCAm, but only 4.3%, 6.5% and 8.1%, respectively, in the spectra of 2Cl4FCAm, 2Cl5FCAm and 2Cl6FCAm. Parallel trends are found in the spectra of BrFCAms and BrClCAms.

In order to compare the competition between the proximity effect and simple cleavages, two further sets of CAms have been investigated, Table 7.

The spectra of 3,4(CH_3_O)_2_CAm and 3,4(OCH_2_O)CAm were obtained to ascertain whether the presence of electron-donating groups in the aromatic ring might favour formation of [M – NH_2_]^+^ and [M – NH_2_ – CO]^+^ at the expense of the proximity effect. However, the RIs of the peaks corresponding to these fragment ions were somewhat lower than those in the spectrum of 4CH_3_OCAm. Furthermore, M^+·^ accounts for the base peak in the spectrum of each of these three CAms containing an OR substituent in position 4; the RI of the signal for [M – H]^+^ is also lower than in the spectra of other CAms in which an electron-withdrawing substituent is present in the 4-position. These trends suggest that the influence of an electron-donating substituent in stabilising the molecular ion outweighs its effect on the stability of the [M – NH_2_]^+^ and [M – NH_2_ – CO]^+^ fragment ions. The reverse trend apparently operates for electron-withdrawing substituents (X = CF_3_ or NO_2_, Table 2).

The spectra of 4(CH_3_)*_n_*CH_3−*n*_CAms (*n* = 1–3) are rather more illuminating. In these cases, simple cleavage of the CH_3_CH_2_, (CH_3_)_2_CH or (CH_3_)_3_C substituent gives rise to [M – CH_3_]^+^. Moreover, the appearance energies for the analogous fragmentations of the corresponding monosubstituted benzenes, 4(CH_3_)*_n_*CH_3−*n*_C_6_H_5_, are known to decrease from 11.3 to 10.7 to 10.3 eV as *n* increases from 1 to 2 to 3,^3^ thus indicating that loss of CH_3_^·^ by simple cleavage of M^+·^ requires less energy as the degree of branching increases. The base peak in the spectrum of 4CH_3_CH_2_CAm corresponds to [M – H]^+^, formed by the proximity effect; the signal for [M – CH_3_]^+^ is much weaker (RI = 13%), but the peak corresponding to [M – NH_2_]^+^ is of moderate RI (29%). On progressing to 4(CH_3_)_2_CHCAm, the peak for [M – H]^+^ remains strong (RI = 66%), but the base peak corresponds to [M – CH_3_]^+^; in addition, the signal for [M – NH_2_]^+^ is weaker (RI = 6.6%). This trend favouring cleavage of the 4-alkyl substituent at the expense of the proximity effect and loss of NH_2_^·^ becomes even more pronounced in the spectrum of 4(CH_3_)_3_CCAm, in which the RIs of the signals for [M – H]^+^, [M – CH_3_]^+^ and [M – NH_2_]^+^ are 6.6%, 100% and <0.5%, respectively. These data indicate that the proximity effect competes far better with simple cleavage of the CH=CHCONH_2_ side chain than with fission of a separate and highly branched side chain in the ‘remote’ 4-position. Furthermore, the huge reduction in the ratio of the RIs of the peaks for [M – H]^+^ and [M – CH_3_]^+^ from 7.7:1 for 4CH_3_CH_2_CAm, to 0.66:1 for 4(CH_3_)_2_CHCAm, to 0.066:1 for 4(CH_3_)_3_CCAm (an overall factor of over 115) emphasises how strongly the competition between the proximity effect and simple cleavage of a branched alkyl substituent varies over a relatively small energy range (of about 1 eV or 23 kcal/mol or 96 kJ/mol). In the present context, the proximity effect competes very effectively with simple cleavage of most unbranched substituents, but care is necessary in interpreting the spectra of CAms containing a branched alkyl substituent, especially a *t-*butyl group.

## Conclusion

Proximity effects are analytically useful in the EI mass spectra of a wide range of substituted cinnamamides. When the X substituent is in the 3-position or 4-position, the signal for [M – H]^+^ typically dominates the spectrum, which displays a weak peak corresponding to [M – X]^+^. In contrast, the signals for [M – X]^+^ generally are prominent in the spectrum of isomeric species in which a range of X (F, Cl, Br, I, CH_3_, CH_3_O, CF_3_ and NO_2_) is in the 2-position. These diagnostic differences permit 2-substituted cinnamamides to be distinguished from their 3-substituted and 4-substituted isomers. Parallel trends are found for disubstituted cinnamamides.

Simple cleavages of CH=CHCONH_2_ side chain rarely compete effectively with the proximity effect in the fragmentation of ionised 2-substituted cinnamamides: the signals for both [M – NH_2_]^+^ and [M – NH_2_ – CO]^+^ in the relevant spectra are much weaker than those for [M – X]^+^ except when X = F or CH_3_. These signals are often more significant in the spectra of the 3-substituted and 4-substituted isomers. In contrast, simple cleavage of a branched 4-alkyl substituent to give [M – CH_3_]^+^ does compete well with formation of [M – H]^+^ by the proximity effect, especially when the substituent is (CH_3_)_3_C.

## Supplemental Material

sj-docx-1-ems-10.1177_14690667231153777 - Supplemental material for Proximity effects in the electron ionisation mass spectra of substituted cinnamamides Click here for additional data file. Supplemental material, sj-docx-1-ems-10.1177_14690667231153777 for Proximity effects in the electron ionisation mass spectra of substituted cinnamamides by Adam R Michalik, Nathan W Fenwick, Richard Telford, Archie W Johnson, William HC Martin and Richard D Bowen in European Journal of Mass Spectrometry
