# Supplementary material for: Proximity effects in the electron ionisation mass spectra of substituted cinnamamides
Source: Eur J Mass Spectrom (Chichester). 2023 Feb 16;29(2):75–87. doi: 10.1177/14690667231153777 (PMC10068410; doi:10.1177/14690667231153777)
Supplement: sj-docx-1-ems-10.1177_14690667231153777 - Supplemental material for Proximity effects in the electron ionisation mass spectra of substituted cinnamamides [file sj-docx-1-ems-10.1177_14690667231153777.docx]

Supplementary Material

Synthesis

All the procedures up to the isolation of the crude substituted cinnamamide were done in a fume cupboard.

General procedure

A mixture of the substituted cinnamic acid (5-20 mmol) and excess thionyl chloride (50-200 mmol) was cautiously heated until a clear solution was obtained (10-30 min). After standing for 1 hr to ensure the reaction was complete, the bulk of the excess thionyl chloride was carefully removed by distillation (bp 74-82 °C) at atmospheric pressure to leave the crude substituted cinnamoyl chloride. This material, if liquid, was added dropwise from a Pasteur pipette during 5-10 min to a magnetically stirred aqueous ammonia solution (specific gravity 0.88, 30-100 mL) covered by ethyl acetate (30-100 ml). If the crude substituted cinnamoyl choride was a solid, it was dissolved in a little ethyl acetate (5-25 ml) before being added to the aqueous ammonia. After allowing the heterogeneous mixture to stir for a further 30-60 min, sufficient distilled water (30-100 mL) was added to permit efficient separation of the two phases. If necessary, more ethyl acetate was added to ensure complete dissolution of the substituted cinnamamide. The upper organic phase was separated and the lower aqueous phase was extracted with ethyl acetate (2 x 30-100 ml). The organic phases were combined, washed with dilute hydrochloric acid (1 M, 10-30 ml), dried (MgSO_4_), filtered and rotary evaporated to constant mass to give the crude substituted cinnamamide, usually as a white to pale cream solid. Recrystallization from ethyl acetate (for the less soluble substituted cinnamic acids) or ethyl acetate and petroleum either (60-80 fraction) gave the pure product. Yields of the crude substituted cinnamamides were generally almost quantitative; yields of recrystallized product ranged from 50-80%.

Specific example.

Application of the above general procedure to a mixture of 4-bromo-2-fluorocinnamic acid (1.25 g, 5.13 mmol) and thionyl chloride (15.0 g, 156 mmol) gave crude 4-bromo-2-fluorocinnamamide (1.22 g, 5.02 mmol, 98%), which was recrystallized from ethyl acetate to give pure product (0.674 g, 27.7 mmol, 54%) as white needles (mp 185-6 °C).

Melting Points

Melting points were determined on a Gallenkamp melting point apparatus. Data are uncorrected.

High Resolution Mass Spectrometry

Accurate molecular mass information was obtained on [M + H]^+^ ions formed in a Orbitrap instrument operating under positive ion electrospray ionization conditions. The calculated (“calcd”) and measured (“found”) m/z values are quoted to five decimal places to facilitate comparison, but the figure in the fifth decimal place may be less reliable than that in the fourth decimal place.

Nuclear Magnetic Resonance Spectrometry

^1^H NMR spectra were obtained on a Bruker instrument operating at 400 MHz. Chemical shift values (δ) are quoted in ppm relative to a value of 0 for (CH_3_)_4_Si. Many signals that are described as “doublets” or “triplets” had fine structure; the chemical shifts for these signals are quoted as a range, as is conventional for multiplets. Coupling constants (*J* values) are quoted in Hz.

CAm

Fine white needles; mp: 150-151 °C (lit: 148 °C)

HRMS (ESI) *m/z* calcd for C_9_H_10_NO^+^: 148.07569 [M + H]^+^; found: 148.075276.

^1^H NMR (400 MHz, CDCl_3_, 25 °C, TMS): δ = 7.65–7.69 (d, *J* = 15.7 Hz, 1H; C=C–H), δ = 7.52–7.57 (m, 2H; Ar–H), δ = 7.38–7.44 (m, 3H; Ar–H), δ = 6.46–6.52 (d, *J* = 15.7 Hz, 1H; C=C–H), δ = 5.58–5.73 (s, 2H; N-H).

2FCAm

Fine white crystals; mp: 137-138 °C (lit: N/A)

HRMS (ESI) *m/z* calcd for C_9_H_9_FNO^+^: 166.06627 [M + H]^+^; found: 166.06602.

^1^H NMR (400 MHz, CDCl_3_, 25 °C, TMS): δ = 7.73–7.77 (d, *J* = 15.8 Hz, 1H; C=C–H), δ = 7.51–7.56 (m, 1H; Ar–H), δ = 7.34–7.40 (m, 1H; Ar–H), δ = 7.16–7.21 (m, 1H; Ar–H), δ = 7.100–7.15 (m, 1H; Ar–H), δ = 6.60–6.64 (d, *J* = 15.8 Hz, 1H; C=C–H), δ = 5.53–5.69 (s, 2H; N-H).

3FCAm

White needles; mp: 126-127 °C (lit: N/A)

HRMS (ESI) *m/z* calcd for C_9_H_9_FNO^+^: 166.06627 [M + H]^+^; found: 166.06606.

^1^H NMR (400 MHz, CDCl_3_, 25 °C, TMS): δ = 7.61–7.65 (d, *J* = 15.6 Hz, 1H; C=C–H), δ = 7.34–7.40 (m, 1H; Ar–H), δ = 7.29–7.31 (m, 1H; Ar–H), δ = 7.21–7.25 (m, 1H; Ar–H), δ = 7.06–7.11 (m, 1H; Ar–H), δ = 6.46–6.50 (d, *J* = 15.6 Hz, 1H; C=C–H), δ = 5.58–5.95 (s, 2H; N-H).

4FCAm

White leaflets; mp: 164-165 °C (lit: N/A)

HRMS (ESI) *m/z* calcd for C_9_H_9_FNO^+^: 166.06627 [M + H]^+^; found: 166.06595.

^1^H NMR (400 MHz, CDCl_3_, 25 °C, TMS): δ = 7.62–7.67 (d, *J* = 15.6 Hz, 1H; C=C–H), δ = 7.50–7.55 (m, 2H; Ar–H), δ = 7.07–7.13 (m, 2H; Ar–H), δ = 6.38–6.42 (d, *J* = 15.6 Hz, 1H; C=C–H), δ = 5.50–5.64 (s, 2H; N-H)

2ClCAm

White crystals; mp: 165-166 °C (lit: 144-146 °C)

HRMS (ESI) *m/z* calcd for C_9_H_9_ClNO^+^: 182.03672 [M + H]^+^; found: 182.03659.

^1^H NMR (400 MHz, CDCl_3_, 25 °C, TMS): δ = 8.01–8.05 (d, *J* = 15.8 Hz, 1H; C=C–H), δ = 7.61–7.63 (dd, *J* = 7.0 Hz, 2.0 Hz, 1H; Ar–H), δ = 7.43–7.46 (dd, *J* = 7.0 Hz, 2.0 Hz, 1H; Ar–H), δ = 7.28–7.35 (m, 2H; Ar–H), δ = 6.46–6.51 (d, *J* = 15.8 Hz, 1H; C=C–H), δ = 5.50–5.69 (s, 2H; N-H).

3ClCAm

Pale cream crystals; mp: 86-88 °C (lit: N/A)

HRMS (ESI) *m/z* calcd for C_9_H_9_ClNO^+^: 182.03672 [M + H]^+^; found: 182.03654.

^1^H NMR (400 MHz, CDCl_3_, 25 °C, TMS): δ = 7.89–7.90 (s, 1H; Ar-H), δ = 7.70–7.73 (m, 1H; Ar–H), δ = 7.54–7.59 (d, *J* = 15.6 Hz, 1H; C=C–H), δ = 7.46–7.49 (m, 1H; Ar–H), δ = 7.12–7.16 (t, *J* = 7.8 Hz, 1H; Ar–H), δ = 6.44–6.48 (d, *J* = 15.6 Hz, 1H; C=C–H), δ = 5.53–5.64 (s, 2H; N-H).

4ClCAm

Fine white leaflets; mp: 214-215 °C (lit: 210-211 °C)

HRMS (ESI) *m/z* calcd for C_9_H_9_NClO^+^: 182.03672 [M + H]^+^; found: 182.03645.

^1^H NMR (400 MHz, CDCl_3_, 25 °C, TMS): δ = 7.61–7.65 (d, *J* = 15.7 Hz, 1H; C=C–H), δ = 7.45–7.49 (d, *J* = 8.7 Hz, 2H; Ar–H), δ = 7.36–7.39 (d, *J* = 8.7 Hz, 2H; Ar–H), δ = 6.43–6.47 (d, *J* = 15.7 Hz, 1H; C=C–H), δ = 5.44–5.58 (s, 2H; N-H).

2BrCAm

Fine white crystals; mp: 181-182 °C (lit: 171-173 °C)

HRMS (ESI) *m/z* calcd for C_9_H_9_BrNO^+^: 225.98620 [M + H]^+^; found: 225.98590.

^1^H NMR (400 MHz, CDCl_3_, 25 °C, TMS): δ = 7.96–8.00 (d, *J* = 15.7 Hz, 1H; C=C–H), δ = 7.63–7.65 (dd, *J* = 8.0 Hz, 1.2 Hz, 1H; Ar–H), δ = 7.59–7.61 (dd, *J* = 8.0 Hz, 1.2 Hz, 1H; Ar–H), δ = 7.32–7.36 (t, *J* = 7.8 Hz, 1H; Ar–H), δ = 7.22–7.26 (t, *J* = 7.8 Hz, 1H; Ar–H), δ = 6.41–6.45 (d, *J* = 15.7 Hz, 1H; C=C–H), δ = 5.55–5.69 (s, 2H; N-H).

3BrCAm

Large white needles; mp: 110-111 °C (lit: N/A)

HRMS (ESI) *m/z* calcd for C_9_H_9_BrNO^+^: 225.98620 [M + H]^+^; found: 225.98582.

^1^H NMR (400 MHz, CDCl_3_, 25 °C, TMS): δ = 7.68–7.69 (s, 1H; Ar-H), δ = 7.58–7.62 (d, *J* = 15.7 Hz, 1H; C=C–H), δ = 7.50–7.53 (m, 1H; Ar–H), δ = 7.43–7.45 (m, 1H; Ar–H), δ = 7.25–7.29 (t, *J* = 7.9 Hz, 1H; Ar–H), δ = 6.46–6.50 (d, *J* = 15.7 Hz, 1H; C=C–H), δ = 5.60–5.81 (s, 2H; N-H).

4BrCAm

Fine white crystals; mp: 217-218 °C (lit: N/A)

HRMS (ESI) *m/z* calcd for C_9_H_9_BrNO^+^: 225.98620 [M + H]^+^; found: 225.98581.

^1^H NMR (400 MHz, CDCl_3_, 25 °C, TMS): δ = 7.50–7.54 (d, *J* = 15.7 Hz, 1H; C=C–H), δ = 7.43–7.46 (d, *J* = 8.5 Hz, 2H; Ar–H), δ = 7.29–7.33 (d, *J* = 8.5 Hz, 2H; Ar–H), δ = 6.35–6.39 (d, *J* = 15.7 Hz, 1H; C=C–H), δ = 5.33–5.53 (s, 2H; N-H).

2ICAm

Very fine pale cream needles; mp: 200-202 °C (lit: 204-205 °C)

HRMS (ESI) *m/z* calcd for C_9_H_9_INO^+^: 273.97233 [M + H]^+^; found: 273.97195.

^1^H NMR (400 MHz, CDCl_3_, 25 °C, TMS): δ = 7.82–7.84 (dd, *J* = 8.0 Hz, 1.2 Hz, 1H; Ar–H), δ = 7.72–8.76 (d, *J* = 15.6 Hz, 1H; C=C–H), δ = 7.45–7.48 (dd, *J* = 8.0 Hz, 1.2 Hz, 1H; Ar–H), δ = 7.26–7.31 (t, *J* = 7.5 Hz, 1H; Ar–H), δ = 6.96–7.00 (t, *J* = 7.5 Hz, 1H; Ar–H), δ = 6.24–6.27 (d, *J* = 15.6 Hz, 1H; C=C–H), δ = 5.38–5.59 (s, 2H; N-H).

3ICAm

White needles; mp: 138-139 °C (lit: N/A)

HRMS (ESI) *m/z* calcd for C_9_H_9_INO^+^: 273.97233 [M + H]^+^; found: 273.97177.

^1^H NMR (400 MHz, CDCl_3_, 25 °C, TMS): δ = 7.89–7.91 (s, 1H; Ar-H), δ = 7.70–7.73 (m, 1H; Ar–H), δ = 7.55–7.59 (d, *J* = 15.7 Hz, 1H; C=C–H), δ = 7.46–7.49 (m, 1H; Ar–H), δ = 7.12–7.16 (t, *J* = 7.8 Hz, 1H; Ar–H), δ = 6.44–6.48 (d, *J* = 15.7 Hz, 1H; C=C–H), δ = 5.53–5.62 (s, 2H; N-H).

4ICAm

mp: 224-225 °C (lit: N/A)

Fine white crystals; HRMS (ESI) *m/z* calcd for C_9_H_9_INO^+^: 273.97233 [M + H]^+^; found: 273.97174.

^1^H NMR (400 MHz, CDCl_3_, 25 °C, TMS): δ = 7.73–7.76 (d, *J* = 8.5 Hz, 2H; Ar–H), δ = 7.58–7.62 (d, *J* = 15.7 Hz, 1H; C=C–H), δ = 7.25–7.28 (d, *J* = 8.5 Hz, 2H; Ar–H), δ = 6.45–6.49 (d, *J* = 15.7 Hz, 1H; C=C–H), δ = 5.42–5.59 (s, 2H; N-H).

2CH_3_CAm

White needles; mp: 157-158 °C (lit: N/A)

HRMS (ESI) *m/z* calcd for C_10_H_12_NO^+^: 162.09134 [M + H]^+^; found: 162.09122.

^1^H NMR (400 MHz, CDCl_3_, 25 °C, TMS): δ = 7.95–7.99 (d, *J* = 15.5 Hz, 1H; C=C–H), δ = 7.53–7.56 (m, 1H; Ar–H), δ = 7.27–7.31 (m, 1H; Ar–H), δ = 7.19–7.24 (m, 2H; Ar–H), δ = 6.37–6.41 (d, *J* = 15.5 Hz, 1H; C=C–H), δ = 5.58–5.68 (s, 2H; N-H), δ = 2.46–2.47 (s, 3H; CH_3_).

3CH_3_CAm

White needles; mp: 96-97 °C (lit: 81-82 °C)

HRMS (ESI) *m/z* calcd for C_10_H_12_NO^+^: 162.09134 [M + H]^+^; found: 162.09116.

^1^H NMR (400 MHz, CDCl_3_, 25 °C, TMS): δ = 7.62–7.66 (d, *J* = 15.7 Hz, 1H; C=C–H), δ = 7.33–7.36 (m, 2H; Ar-H), δ = 7.27–7.31 (t, *J* = 7.49 Hz, 1H; Ar–H), δ = 7.19–7.22 (m, 1H; Ar–H), δ = 6.45–6.50 (d, *J* = 15.7 Hz, 1H; C=C–H), δ = 5.61–5.82 (s, 2H; N-H) , δ = 2.38–2.40 (s, 3H; CH_3_).

4CH_3_CAm

White leaflets; mp: 190-191 °C (lit: 187.5 °C)

HRMS (ESI) *m/z* calcd for C_10_H_12_NO^+^: 162.09134 [M + H]^+^; found: 162.09105.

^1^H NMR (400 MHz, CDCl_3_, 25 °C, TMS): δ = 7.53–7.58 (d, *J* = 15.7 Hz, 1H; C=C–H), δ = 7.33–7.36 (d, *J* = 8.2 Hz, 2H; Ar–H), δ = 7.11–7.13 (d, *J* = 8.2 Hz, 2H; Ar–H), δ = 6.32–6.36 (d, *J* = 15.7 Hz, 1H; C=C–H), δ = 5.41–5.53 (s, 2H; N-H), δ = 2.29–2.32 (s, 3H; CH_3_).

2CH_3_OCAm

Fine pale cream needles; mp: 196-197 °C (lit: 189-191 °C)

HRMS (ESI) *m/z* calcd for C_10_H_12_NO_2_^+^: 178.08626 [M + H]^+^; found: 178.08609.

^1^H NMR (400 MHz, CDCl_3_, 25 °C, TMS): δ = 7.90–7.94 (d, *J* = 15.7 Hz, 1H; C=C–H), δ = 7.50–7.53 (dd, *J* = 7.7 Hz, 1.7 Hz, 1H; Ar–H), δ = 7.34–7.38 (m, 1H; Ar–H), δ = 6.97–7.01 (t, *J* = 7.7 Hz, 1H; Ar–H), δ = 6.93–6.96 (d, *J* = 8.6 Hz, 1H; Ar–H), δ = 6.57–6.35 (d, *J* = 15.7 Hz, 1H; C=C–H), δ = 5.38–5.62 (s, 2H; N-H), δ = 3.91–3.92 (s, 3H; OCH_3_).

3CH_3_OCAm

Fine pale cream crystals; mp: 126-127 °C (lit: N/A)

HRMS (ESI) *m/z* calcd for C_10_H_12_NO_2_^+^: 178.08626 [M + H]^+^; found: 178.08600.

^1^H NMR (400 MHz, CDCl_3_, 25 °C, TMS): δ = 7.61–7.66 (d, *J* = 15.7 Hz, 1H; C=C–H), δ = 7.30–7.34 (t, *J* = 7.9 Hz, 1H; Ar–H), δ = 7.12–7.15 (m, 1H; Ar–H), δ = 7.05–7.07 (s, 1H; Ar-H), δ = 6.93–6.96 (m, 1H; Ar–H), δ = 6.44–6.49 (d, *J* = 15.7 Hz, 1H; C=C–H), δ = 5.53–5.65 (s, 2H; N-H), δ = 3.85–3.86 (s, 3H; OCH_3_).

4CH_3_OCAm

Very fine white needles; mp: 194-195 °C (lit: 199-201 °C)

HRMS (ESI) *m/z* calcd for C_10_H_12_NO_2_^+^: 178.08626 [M + H]^+^; found: 178.08601.

^1^H NMR (400 MHz, CDCl_3_, 25 °C, TMS): δ = 7.51–7.56 (d, *J* = 15.7 Hz, 1H; C=C–H), δ = 7.38–7.42 (d, *J* = 8.8 Hz, 2H; Ar–H), δ = 6.81–6.85 (d, *J* = 8.8 Hz, 2H; Ar–H), δ = 6.23–6.28 (d, *J* = 15.7 Hz, 1H; C=C–H), δ = 5.35–5.47 (s, 2H; N-H) ), δ = 3.76–3.78 (s, 3H; OCH_3_).

2CF_3_CAm

Fine white crystals; mp: 182-183 °C (lit: N/A)

HRMS (ESI) *m/z* calcd for C_10_H_9_F_3_NO^+^: 216.06308 [M + H]^+^; found: 216.06242.

^1^H NMR (400 MHz, CDCl_3_, 25 °C, TMS): δ = 7.96–8.02 (d, *J* = 15.7 Hz, 1H; C=C–H), δ = 7.72–7.74 (d, *J* = 7.7 Hz, 1H; Ar–H), δ = 7.68–7.71 (d, *J* = 7.7 Hz, 1H; Ar–H), δ = 7.56–7.61 (t, *J* = 7.6 Hz, 1H; Ar–H), δ = 7.47–7.52 (t, *J* = 7.6 Hz, 1H; Ar–H), δ = 6.41–6.46 (d, *J* = 15.7 Hz, 1H; C=C–H), δ = 5.58–5.68 (s, 2H; N-H).

3CF_3_CAm

Fine white crystals; mp: 103-104 °C (lit: N/A)

HRMS (ESI) *m/z* calcd for C_10_H_9_F_3_NO ^+^: 216.06308 [M + H]^+^; found: 216.06215.

^1^H NMR (400 MHz, CDCl_3_, 25 °C, TMS): δ = 7.79–7.80 (s, 1H; Ar-H), δ = 7.68–7.73 (d, *J* = 15.7 Hz, 1H; C=C–H), δ = 7.68–7.71 (m, 1H; Ar–H), δ = 7.63–7.66 (m, 1H; Ar–H), δ = 7.52–7.56 (t, *J* = 8.0 Hz, 1H; Ar–H), δ = 6.53–6.57 (d, *J* = 15.7 Hz, 1H; C=C–H), δ = 5.57–5.65 (s, 2H; N-H).

4CF_3_CAm

Large white plates; mp: 181-182 °C (lit: N/A)

HRMS (ESI) *m/z* calcd for C_10_H_9_F_3_NO ^+^: 216.06308 [M + H]^+^; found: 216.06213.

^1^H NMR (400 MHz, CDCl_3_, 25 °C, TMS): δ = 7.68–7.72 (d, *J* = 15.8 Hz, 1H; C=C–H), δ = 7.62–7.68 (m, 4H; Ar–H), δ = 6.53–6.57 (d, *J* = 15.8 Hz, 1H; C=C–H), δ = 5.55–5.62 (s, 2H; N-H).

2NO_2_CAm

Fine cream needles; mp: 185-186 °C (lit: 186-186.5 °C)

HRMS (ESI) *m/z* calcd for C_9_H_9_N_2_O_3_^+^: 193.06077 [M + H]^+^; found: 193.06041.

^1^H NMR (400 MHz, CDCl_3_, 25 °C, TMS): δ = 8.06–8.09 (dd, *J* = 8.1 Hz, 1.4 Hz, 1H; Ar–H), δ = 8.00–8.05 (d, *J* = 15.8 Hz, 1H; C=C–H), δ = 7.54–7.68 (m, 3H; Ar–H), δ = 6.37–6.41 (d, *J* = 15.8 Hz, 1H; C=C–H), δ = 5.48–5.66 (s, 2H; N-H).

3NO_2_CAm

Cream needles; mp: 199-200 °C (lit: 195-196 °C)

HRMS (ESI) *m/z* calcd for C_9_H_9_N_2_O_3_^+^: 193.06077 [M + H]^+^; found: 193.06036.

^1^H NMR (400 MHz, CDCl_3_, 25 °C, TMS): δ = 8.41–8.44 (s, 1H; Ar-H), δ = 8.23–8.27 (m, 1H; Ar–H), δ = 7.81–7.84 (m, 1H; Ar–H), δ = 7.71–7.76 (d, *J* = 15.7 Hz, 1H; C=C–H), δ = 7.59–7.63 (t, *J* = 8.0 Hz, 1H; Ar–H), δ = 6.59–6.64 (d, *J* = 15.7 Hz, 1H; C=C–H), δ = 5.51–5.68 (s, 2H; N-H).

4NO_2_CAm

Fine pale yellow leaflets; mp: 220-221 °C (lit: 217 °C)

HRMS (ESI) *m/z* calcd for C_9_H_9_N_2_O_3_^+^: 193.06077 [M + H]^+^; found: 193.06009.

^1^H NMR (400 MHz, CDCl_3_, 25 °C, TMS): δ = 8.17–8.21 (d, *J* = 8.7 Hz, 2H; Ar–H), δ = 7.62–7.66 (d, *J* = 15.7 Hz, 1H; C=C–H), δ = 7.58–7.61 (d, *J* = 8.7 Hz, 2H; Ar–H), δ = 6.48–6.53 (d, *J* = 15.7 Hz, 1H; C=C–H), δ = 5.45–5.55 (s, 2H; N-H).

2,3Cl_2_CAm

Fine white crystals; mp: 182-183 °C (lit: N/A)

HRMS (ESI) *m/z* calcd for C_9_H_8_Cl_2_NO^+^: 215.99775 [M + H]^+^; found: 216.06271.

^1^H NMR (400 MHz, CDCl_3_, 25 °C, TMS): δ = 8.01–8.05 (d, *J* = 15.7 Hz, 1H; C=C–H), δ = 7.49–7.52 (m, 2H; Ar–H), δ = 7.22–7.26 (t, J = 8.0 Hz, 1H; Ar–H), δ = 6.43-6.48 (d, *J* = 15.7 Hz, 1H; C=C–H), δ = 5.54–5.69 (s, 2H; N-H).

2,4Cl_2_CAm

White leaflets; mp: 179-180 °C (lit: N/A)

HRMS (ESI) *m/z* calcd for C_9_H_8_Cl_2_NO^+^: 215.99775 [M + H]^+^; found: 215.99734.

^1^H NMR (400 MHz, CDCl_3_, 25 °C, TMS): δ = 7.94–7.98 (d, *J* = 15.8 Hz, 1H; C=C–H), δ = 7.54–7.55 (d, *J* = 8.4 Hz, 1H; Ar–H), δ = 7.46–7.47 (d, J = 2.1 Hz, 1H; Ar–H), δ = 7.27–7.29 (m, 1H; Ar–H), δ = 6.43-6.49 (d, *J* = 15.8 Hz, 1H; C=C–H), δ = 5.55–5.67 (s, 2H; N-H).

2,5Cl_2_CAm

Fine white needles; mp: 205-206 °C (lit: N/A)

HRMS (ESI) *m/z* calcd for C_9_H_8_Cl_2_NO^+^: 215.99775 [M + H]^+^; found: 215.99736.

^1^H NMR (400 MHz, CDCl_3_, 25 °C, TMS): δ = 7.94–7.98 (d, *J* = 15.8 Hz, 1H; C=C–H), δ = 7.59–7.60 (s, 1H; Ar–H), δ = 7.37–7.39 (d, *J* = 8.7 Hz, 1H; Ar–H), δ = 7.28–7.31 (dd, *J* = 8.7 Hz, 2.4 Hz, 1H; Ar–H), δ = 6.45–6.49 (d, *J* = 15.8 Hz, 1H; C=C–H), δ = 5.49–5.64 (s, 2H; N-H).

2,6Cl_2_CAm

Very fine white needles; mp: 165-166 °C (lit: 179-180 °C)

HRMS (ESI) *m/z* calcd for C_9_H_8_Cl_2_NO^+^: 215.99775 [M + H]^+^; found: 215.99738.

^1^H NMR (400 MHz, CDCl_3_, 25 °C, TMS): δ = 7.73–7.78 (d, *J* = 16.0 Hz, 1H; C=C–H), δ = 7.36–7.39 (d, *J* = 8.1 Hz, 2H; Ar–H), δ = 7.18–7.23 (t, *J* = 8.1 Hz, 1H; Ar–H), δ = 6.61–6.66 (d, *J* = 16.0 Hz, 1H; C=C–H), δ = 5.59–5.75 (s, 2H; N-H).

3,4Cl_2_CAm

Very fine white needles; mp: 139-140 °C (lit: N/A)

HRMS (ESI) *m/z* calcd for C_9_H_8_Cl_2_NO^+^: 215.99775 [M + H]^+^; found: 215.99727.

^1^H NMR (400 MHz, CDCl_3_, 25 °C, TMS): δ = 7.61–7.63 (s, 1H; Ar–H), δ = 7.55–7.60 (d, *J* = 15.7 Hz, 1H; C=C–H), δ = 7.46–7.49 (d, *J* = 8.3 Hz, 2H; Ar–H), δ = 7.33–7.36 (d, *J* = 8.3 Hz, 1H; Ar–H), δ = 6.44–6.49 (d, *J* = 15.7 Hz, 1H; C=C–H), δ = 5.59–5.77 (s, 2H; N-H).

3,5Cl_2_CAm

Very fine white needles; mp: 158-159 °C (lit: N/A)

HRMS (ESI) *m/z* calcd for C_9_H_8_Cl_2_NO^+^: 215.99775 [M + H]^+^; found: 215.99724.

^1^H NMR (400 MHz, CDCl_3_, 25 °C, TMS): δ = 7.53–7.58 (d, *J* = 15.6 Hz, 1H; C=C–H), δ = 7.37–7.41 (m, 3H; Ar–H), δ = 6.46–6.50 (d, *J* = 15.6 Hz, 1H; C=C–H), δ = 5.51–5.62 (s, 2H; N-H).

2Cl4FCAm

Fine white leaflets; mp: 187-188 °C (lit: 177-179 °C)

HRMS (ESI) *m/z* calcd for C_9_H_8_ClFNO^+^: 200.02730 [M + H]^+^; found: 200.02676.

^1^H NMR (400 MHz, CDCl_3_, 25 °C, TMS): δ = 8.12–8.17 (d, *J* = 15.7 Hz, 1H; C=C–H), δ = 7.65–7.69 (m, 1H; Ar–H), δ = 7.21–7.24 (m, 1H; Ar–H), δ = 6.99–7.04 (m, 1H; Ar–H), δ = 6.40–6.44 (d, *J* = 15.7 Hz, 1H; C=C–H), δ = 4.89–4.94 (s, 2H; N-H).

2Cl5FCAm

Fine white needles; mp: 167-168 °C (lit: 158-159 °C)

HRMS (ESI) *m/z* calcd for C_9_H_8_ClFNO^+^: 200.02730 [M + H]^+^; found: 200.02687.

^1^H NMR (400 MHz, CDCl_3_, 25 °C, TMS): δ = 7.95–7.99 (d, *J* = 15.8 Hz, 1H; C=C–H), δ = 7.39–7.43 (dd, *J* = 8.8 Hz, 5.1 Hz, 1H; Ar–H), δ = 7.29–7.33 (dd, J = 8.8 Hz, 3.0 Hz, 1H; Ar–H), δ = 7.02–7.08 (m, 1H; Ar–H), δ = 6.43-6.48 (d, *J* = 15.8 Hz, 1H; C=C–H), δ = 5.63–5.72 (s, 2H; N-H).

2Cl6FCAm

Fine pale cream crystals; mp: 130-131 °C (lit: N/A)

HRMS (ESI) *m/z* calcd for C_9_H_8_ClFNO^+^: 200.02730 [M + H]^+^; found: 200.02684.

^1^H NMR (400 MHz, CDCl_3_, 25 °C, TMS): δ = 7.88–7.93 (d, *J* = 15.9 Hz, 1H; C=C–H), δ = 7.25–7.29 (m, 2H; Ar–H), δ = 7.04–7.09 (m, 1H; Ar–H), δ = 6.74–6.79 (d, *J* = 15.9 Hz, 1H; C=C–H), δ = 5.68–5.81 (s, 2H; N-H).

3Cl5FCAm

Very fine white needles; mp: 151-152 °C (lit: N/A)

HRMS (ESI) *m/z* calcd for C_9_H_8_ClFNO^+^: 200.02730 [M + H]^+^; found: 200.02678.

^1^H NMR (400 MHz, CDCl_3_, 25 °C, TMS): δ = 7.54–7.59 (d, J = 15.7 Hz, 1H, C=C-H), δ = 7.31–7.32 (s, 1H; Ar–H), δ = 7.10–7.15 (m, 2H; Ar–H), δ = 6.45–6.50 (d, *J* = 15.7 Hz, 1H; C=C–H), δ = 5.54–5.65 (s, 2H; N-H).

3Cl6FCAm

Fine white needles; mp: 156-157 °C (lit: N/A)

HRMS (ESI) *m/z* calcd for C_9_H_8_ClFNO^+^: 200.02730 [M + H]^+^; found: 200.02675.

^1^H NMR (400 MHz, CDCl_3_, 25 °C, TMS): δ = 7.57–7.62 (d, *J* = 15.9 Hz, 1H; C=C–H), δ = 7.40–7.43 (m, 1H; Ar–H), δ = 7.20–7.25 (m, 1H; Ar–H), δ = 6.96–7.01 (m, 1H; Ar–H), δ = 6.48-6.53 (d, *J* = 15.9 Hz, 1H; C=C–H), δ = 5.51–5.60 (s, 2H; N-H).

4Cl2FCAm

Fine white leaflets; mp: 171-172 °C (lit: N/A)

HRMS (ESI) *m/z* calcd for C_9_H_8_ClFNO^+^: 200.02730 [M + H]^+^; found: 200.02681.

^1^H NMR (400 MHz, CDCl_3_, 25 °C, TMS): δ = 7.84–7.89 (d, *J* = 15.7 Hz, 1H; C=C–H), δ = 7.50–7.55 (m, 1H; Ar–H), δ = 7.17–7.23 (m, 2H; Ar–H), δ = 6.53–6.59 (d, *J* = 15.7 Hz, 1H; C=C–H), δ = 4.90–4.96 (s, 2H; N-H).

2Br4FCAm

Fine white needles; mp: 190-191 °C (lit: N/A)

HRMS (ESI) *m/z* calcd for C_9_H_8_BrFNO^+^: 243.97678 [M + H]^+^; found: 243.97627.

^1^H NMR (400 MHz, CDCl_3_, 25 °C, TMS): δ = 7.91–7.95 (d, *J* = 15.7 Hz, 1H; C=C–H), δ = 7.57–7.61 (dd, *J* = 8.3 Hz, 5.9 Hz, 1H; Ar–H), δ = 7.38–7.41 (dd, *J* = 8.3 Hz, 2.6 Hz, 1H; Ar–H), δ = 7.05–7.11 (m, 1H; Ar–H), δ = 6.34–6.40 (d, *J* = 15.7 Hz, 1H; C=C–H), δ = 5.46–5.63 (s, 2H; N-H).

2Br5FCAm

Large translucent white plates; mp: 161-162 °C (lit: N/A)

HRMS (ESI) *m/z* calcd for C_9_H_8_BrFNO^+^: 243.97678 [M + H]^+^; found: 243.97650.

^1^H NMR (400 MHz, CDCl_3_, 25 °C, TMS): δ = 7.90–7.95 (d, *J* = 15.7 Hz, 1H; C=C–H), δ = 7.58–7.61 (dd, *J* = 8.9 Hz, 5.9 Hz, 1H; Ar–H), δ = 7.29–7.32 (dd, *J* = 8.9 Hz, 3.0 Hz, 1H; Ar–H), δ = 6.96–7.01 (m, 1H; Ar–H), δ = 6.39–6.43 (d, *J* = 15.7 Hz, 1H; C=C–H), δ = 5.56–5.69 (s, 2H; N-H).

3Br5FCAm

Fine white leaflets; mp: 133-134 °C (lit: N/A)

HRMS (ESI) *m/z* calcd for C_9_H_8_BrFNO^+^: 243.97678 [M + H]^+^; found: 243.97635.

^1^H NMR (400 MHz, CDCl_3_, 25 °C, TMS): δ = 7.54–7.58 (d, J = 15.7 Hz, 1H, C=C-H), δ = 7.46–7.47 (s, 1H; Ar–H), δ = 7.25–7.28 (m, 1H; Ar–H), δ = 7.14–7.18 (m, 1H; Ar–H), δ = 6.45–6.49 (d, *J* = 15.7 Hz, 1H; C=C–H), δ = 5.60–5.88 (s, 2H; N-H).

3Br6FCAm

Very fine white needles; mp: 154-155 °C (lit: N/A)

HRMS (ESI) *m/z* calcd for C_9_H_8_BrFNO^+^: 243.97678 [M + H]^+^; found: 243.97644.

^1^H NMR (400 MHz, CDCl_3_, 25 °C, TMS): δ = 7.66–7.70 (d, *J* = 15.7 Hz, 1H; C=C–H), δ = 7.64–7.66 (m, 1H; Ar–H), δ = 7.44–7.48 (m, 1H; Ar–H), δ = 7.00–7.05 (m, 1H; Ar–H), δ = 6.57-6.61 (d, *J* = 15.7 Hz, 1H; C=C–H), δ = 5.58–5.71 (s, 2H; N-H).

4Br2FCAm

Fine white leaflets; mp: 185-186 °C (lit: N/A)

HRMS (ESI) *m/z* calcd for C_9_H_8_BrFNO^+^: 243.97678 [M + H]^+^; found: 243.97636.

^1^H NMR (400 MHz, CDCl_3_, 25 °C, TMS): δ = 7.55–7.60 (d, *J* = 15.9 Hz, 1H; C=C–H), δ = 7.21–7.34 (m, 4H; Ar–H), δ = 6.49–6.54 (d, *J* = 15.9 Hz, 1H; C=C–H), δ = 5.48–5.54 (s, 2H; N-H).

2Br4ClCAm

Very fine white needles; mp: 185-186 °C (lit: N/A)

HRMS (ESI) *m/z* calcd for C_9_H_8_BrClNO^+^: 259.94723 [M + H]^+^; found: 259.94684.

^1^H NMR (400 MHz, CDCl_3_, 25 °C, TMS): δ = 7.90–7.95 (d, *J* = 15.7 Hz, 1H; C=C–H), δ = 7.65–7.66 (d, *J* = 2.1 Hz, 1H; Ar–H), δ = 7.51–7.54 (d, *J* = 8.5 Hz, 1H; Ar–H), δ = 7.32–7.34 (dd, *J* = 8.5 Hz, 2.1 Hz, 1H; Ar–H), δ = 6.39–6.43 (d, *J* = 15.7 Hz, 1H; C=C–H), δ = 5.51–5.67 (s, 2H; N-H).

2Br5ClCAm

Very fine white needles; mp: 198-199 °C (lit: N/A)

HRMS (ESI) *m/z* calcd for C_9_H_8_BrClNO^+^: 259.94723 [M + H]^+^; found: 259.94711.

^1^H NMR (400 MHz, CDCl_3_, 25 °C, TMS): δ = 7.80–7.85 (d, *J* = 15.7 Hz, 1H; C=C–H), δ = 7.48–7.49 (s, 1H; Ar–H), δ = 7.46–7.48 (d, *J* = 8.6 Hz, 1H; Ar–H), δ = 7.11–7.14 (d, *J* = 8.6 Hz, 1H; Ar–H), δ = 6.31–6.35 (d, *J* = 15.7 Hz, 1H; C=C–H), δ = 5.43–5.56 (s, 2H; N-H).

2Br6ClCAm

Very fine white needles; mp: 179-180 °C (lit: N/A)

HRMS (ESI) *m/z* calcd for C_9_H_8_BrClNO^+^: 259.94723 [M + H]^+^; found: 259.94693.

^1^H NMR (400 MHz, CDCl_3_, 25 °C, TMS): δ = 7.66–7.71 (d, *J* = 15.9 Hz, 1H; C=C–H), δ = 7.56–7.58 (d, *J* = 8.0 Hz, 1H; Ar–H), δ = 7.41–7.43 (d, *J* = 8.0 Hz, 1H; Ar–H), δ = 7.10–7.15 (t, *J* = 8.0 Hz, 1H; Ar–H), δ = 6.51–6.55 (d, *J* = 15.9 Hz, 1H; C=C–H), δ = 5.51–5.67 (s, 2H; N-H).

3Br5ClCAm

Very fine white needles; mp: 167-168 °C (lit: N/A)

HRMS (ESI) *m/z* calcd for C_9_H_8_BrClNO^+^: 259.94723 [M + H]^+^; found: 259.94705.

^1^H NMR (400 MHz, CDCl_3_, 25 °C, TMS): δ = 7.52–7.57 (m, 3H), δ = 7.43–7.45 (s, 1H; Ar–H), δ = 6.45–6.50 (d, *J* = 15.7 Hz, 1H; C=C–H), δ = 5.59–5.68 (s, 2H; N-H).

4Br2ClCAm

White leaflets; mp: 183-184 °C (lit: N/A)

HRMS (ESI) *m/z* calcd for C_9_H_8_BrClNO^+^: 259.94723 [M + H]^+^; found: 259.94693.

^1^H NMR (400 MHz, CDCl_3_, 25 °C, TMS): δ = 7.92–7.97 (d, *J* = 15.7 Hz, 1H; C=C–H), δ = 7.62–7.64 (d, *J* = 1.9 Hz, 1H; Ar–H), δ = 7.46–7.49 (d, *J* = 8.4 Hz, 1H; Ar–H), δ = 7.42–7.45 (dd, *J* = 8.4 Hz, 1.9 Hz, 1H; Ar–H), δ = 6.45–6.49 (d, *J* = 15.7 Hz, 1H; C=C–H), δ = 5.23–5.63 (s, 2H; N-H).

5Br2ClCAm

Fine white needles; mp: 194-195 °C lit: N/A)

HRMS (ESI) *m/z* calcd for C_9_H_8_BrClNO^+^: 259.94723 [M + H]^+^; found: 259.94711.

^1^H NMR (400 MHz, CDCl_3_, 25 °C, TMS): δ = 7.84–7.88 (d, *J* = 15.7 Hz, 1H; C=C–H), δ = 7.64–7.66 (s, 1H; Ar–H), δ = 7.33–7.36 (d, *J* = 8.7 Hz, 1H; Ar–H), δ = 7.21–7.23 (d, *J* = 8.7 Hz, 1H; Ar–H), δ = 6.35–6.39 (d, *J* = 15.7 Hz, 1H; C=C–H), δ = 5.49–5.60 (s, 2H; N-H).

3,4(CH_3_O)_2_CAm

Fine white crystals; mp: 168-169 °C (lit: 164-166 °C)

HRMS (ESI) *m/z* calcd for C_11_H_14_NO_3_^+^: 208.09682 [M + H]^+^; found: 208.09607.

^1^H NMR (400 MHz, CDCl_3_, 25 °C, TMS): δ = 7.58–7.63 (d, *J* = 15.7 Hz, 1H; C=C–H), δ = 7.11–7.14 (d, *J* = 8.2 Hz, 1H; Ar–H), δ = 7.05–7.07 (s, 1H; Ar–H), δ = 6.87–6.90 (d, *J* = 8.2 Hz, 1H; Ar–H), δ = 6.33–6.38 (d, *J* = 15.7 Hz, 1H; C=C–H), δ = 5.55–5.65 (s, 2H; N-H), δ = 3.92–3.95 (s, 6H; OCH_3_).

3,4(OCH_2_O)CAm

Pale cream needles; mp: 185-186 °C (lit: 182 °C)

HRMS (ESI) *m/z* calcd for C_10_H_10_NO_3_^+^: 192.06552 [M + H]^+^; found: 192.06500.

^1^H NMR (400 MHz, CDCl_3_, 25 °C, TMS): δ = 7.57–7.61 (d, *J* = 15.6 Hz, 1H; C=C–H), δ = 7.01–7.05 (m, 2H; Ar–H), δ = 6.82–6.85 (d, *J* = 7.9 Hz 1H; Ar–H), δ = 6.27–6.33 (d, *J* = 15.6 Hz, 1H; C=C–H), δ = 6.02–6.03 (s, 2H; OCH_2_O), δ = 5.39–5.52 (s, 2H; N-H).

4CH_3_CH_2_CAm

Fine white crystals; mp: 173-174 °C (lit: N/A)

HRMS (ESI) *m/z* calcd for C_11_H_14_NO^+^: 176.10699 [M + H]^+^; found: 176.10693.

^1^H NMR (400 MHz, CDCl_3_, 25 °C, TMS): δ = 7.63–7.67 (d, *J* = 15.6 Hz, 1H; C=C-H), δ = 7.44–7.48 (d, *J* = 8.1 Hz, 2H; Ar–H), δ = 7.21–7.25 (d, *J* = 8.1 Hz, 2H; Ar–H), δ = 6.42–6.47 (d, *J* = 15.6 Hz, 1H; C=C–H), δ = 5.60–5.75 (s, 2H; N-H), δ = 2.65–2.73 (q, *J* = 7.6 Hz, 2H; CH_2_), δ = 1.24–1.29 (t, *J* = 7.6 Hz, 3H; CH_3_).

4(CH_3_)_2_CHCAm

Pale cream crystals; mp: 182-183 °C (lit: N/A)

HRMS (ESI) *m/z* calcd for C_12_H_16_NO^+^: 190.12264 [M + H]^+^; found: 190.12221.

^1^H NMR (400 MHz, CDCl_3_, 25 °C, TMS): δ = 7.63–7.68 (d, *J* = 15.8 Hz, 1H; C=C-H), δ = 7.46–7.49 (d, *J* = 8.2 Hz, 2H; Ar–H), δ = 7.25–7.28 (d, *J* = 8.2 Hz, 2H; Ar–H), δ = 6.41–6.46 (d, *J* = 15.8 Hz, 1H; C=C–H), δ = 5.52–5.61 (s, 2H; N-H), δ = 2.89–3.00 (m, 1H; CH), δ = 1.25–1.30 (d, *J* = 6.9 Hz, 6H; CH_3_).

4(CH_3_)_3_CCAm

White leaflets; mp: 186-187 °C (lit: N/A)

HRMS (ESI) *m/z* calcd for C_13_H_18_NO^+^: 204.13829 [M + H]^+^; found: 204.13779.

^1^H NMR (400 MHz, CDCl_3_, 25 °C, TMS): δ = 7.64–7.68 (d, *J* = 15.7 Hz, 1H; C=C-H), δ = 7.47–7.50 (d, *J* = 8.3 Hz, 2H; Ar–H), δ = 7.41–7.44 (d, *J* = 8.3 Hz, 2H; Ar–H), δ = 6.42–6.47 (d, *J* = 15.7 Hz, 1H; C=C–H), δ = 5.55–5.63 (s, 2H; N-H), δ = 1.33–1.37 (s, 9H; CH_3_).
